# Structural Basis of Antibody Conformation and Stability Modulation by Framework Somatic Hypermutation

**DOI:** 10.3389/fimmu.2021.811632

**Published:** 2022-01-03

**Authors:** Zizhang Sheng, Jude S. Bimela, Phinikoula S. Katsamba, Saurabh D. Patel, Yicheng Guo, Haiqing Zhao, Youzhong Guo, Peter D. Kwong, Lawrence Shapiro

**Affiliations:** ^1^ Zuckerman Mind Brain Behavior Institute, Columbia University, New York, NY, United States; ^2^ Aaron Diamond AIDS Research Center, Columbia University Vagelos College of Physicians and Surgeons, New York, NY, United States; ^3^ Department of Systems Biology, Columbia University, New York, NY, United States; ^4^ Department of Medicinal Chemistry, Virginia Commonwealth University, Richmond, VA, United States; ^5^ Institute for Structural Biology, Drug Discovery, and Development, Virginia Commonwealth University, Richmond, VA, United States; ^6^ Vaccine Research Center, National Institute of Allergy and Infectious Diseases, National Institutes of Health, Bethesda, MD, United States; ^7^ Department of Biochemistry and Molecular Biophysics, Columbia University, New York, NY, United States

**Keywords:** antibody, broadly HIV-1 neutralizing antibody, conformation modulation, epistasis, INDEL, molecular dynamics simulation, somatic hypermutation, stability

## Abstract

Accumulation of somatic hypermutation (SHM) is the primary mechanism to enhance the binding affinity of antibodies to antigens *in vivo*. However, the structural basis of the effects of many SHMs remains elusive. Here, we integrated atomistic molecular dynamics (MD) simulation and data mining to build a high-throughput structural bioinformatics pipeline to study the effects of individual and combination SHMs on antibody conformation, flexibility, stability, and affinity. By applying this pipeline, we characterized a common mechanism of modulation of heavy-light pairing orientation by frequent SHMs at framework positions 39_H_, 91_H_, 38_L_, and 87_L_ through disruption of a conserved hydrogen-bond network. Q39L_H_ alone and in combination with light chain framework 4 (FWR4_L_) insertions further modulated the elbow angle between variable and constant domains of many antibodies, resulting in improved binding affinity for a subset of anti-HIV-1 antibodies. Q39L_H_ also alleviated aggregation induced by FWR4_L_ insertion, suggesting remote epistasis between these SHMs. Altogether, this study provides tools and insights for understanding antibody affinity maturation and for engineering functionally improved antibodies.

## Introduction

The affinity maturation process of antibodies or B cell receptors (BCRs) constitutes a microevolution system for antibody improvement (1). During affinity maturation, multiple types of somatic hypermutations (SHMs) (point mutations, insertions and deletions (indels), and sites for post-translational modifications) are incorporated in the BCR variable domain (2, 3). Beneficial SHMs are selected iteratively to optimize the properties of BCRs including antigen-binding affinity as well as the accommodation of antigen variability, flexibility, and physical stability (4–10). In previous studies (11, 12), we built gene-specific substitution profiles (GSSPs) to describe gene-specific hotspots and preferences of point SHMs. We found that SHMs are generated with strong preferences resulting in frequent or dominant convergent mutations, which are commonly observed amongst different antibody lineages (12, 13). Nevertheless, the functional impact and mechanistic basis of numerous SHMs and their combinations remain poorly understood. As mapping the development of functionally important antibody lineages has become commonplace, the need for a “dictionary” to interpret these developmental maps has become clear.

Structurally, the complementarity-determining regions (CDRs) from both heavy and light chains form a paratope, while framework regions (FWRs) of each chain form a 2-layered β-sandwich to present and stabilize the conformations of CDRs (14). SHMs in CDRs undergo frequent antigen-specific selection to optimize the physical non-covalent interactions between paratope and epitope. FWRs are more conserved than CDRs; however, new evidence demonstrates the critical roles of FWR SHMs in both *in vivo* and *in vitro* affinity maturation (15–18). In contrast to SHMs in CDRs, many beneficial SHMs in FWRs modulate antibody features remotely by altering the stability and conformations of CDRs, the pairing of specific V_H_-V_L_ interactions, and the elbow angles between the variable and constant domains (V_H_-C_H_1 or V_L_-C_L_) (15, 18–23). Because many FWR residues are conserved among germline genes, a FWR SHM could affect antibody features consistently among antibodies with different gene origins, and we thus refer to such consistent affects as a common mechanism of modulation. For example, Koenig et al. showed that dominant SHMs at light chain position 83 [Kabat numbering (24)] alter the elbow angle and V_H_-V_L_ angle in many antibodies, resulting in changes in antigen-binding affinity and stability (20).

Currently, approaches where structure determination is coupled to biophysical readouts [e.g. X-ray crystallography with surface plasmon resonance (SPR)] are used to characterize mechanisms of affinity improvement by SHMs. However, such approaches are time-consuming and expensive, and it is impractical to undertake such detailed experimental studies to characterize the effects of SHMs in all cases. With the development of high-performance graphics processing unit (GPU) computers, molecular dynamics (MD) simulations have proven effective at evaluating structural alterations by SHMs (25–28). In addition, about 6000 antibody structures are available in the Protein Data Bank (PDB), which form a valuable informative dataset to examine the effects of SHMs on antibody structure. A bioinformatics platform to interrogate this information could provide a fast and low-cost method, complementary to experimental approaches, for understanding the functions of SHMs and the process of antibody-affinity maturation.

In this study, we integrated MD simulation and a non-redundant antibody structure database to investigate SHM-induced conformation changes. We also applied SPR, thermostability measurement, and dynamic light scattering to evaluate the effects of SHMs on antigen-binding affinity, stability, and aggregation propensity respectively. We found a common mechanism of V_H_-V_L_ conformation modulation by V_H_-V_L_ interface and elbow interface SHMs and characterized epistasis effects between these SHMs.

## Results

### A Structural Bioinformatic Pipeline to Interrogate the Effects of SHM

**Figure 1:**
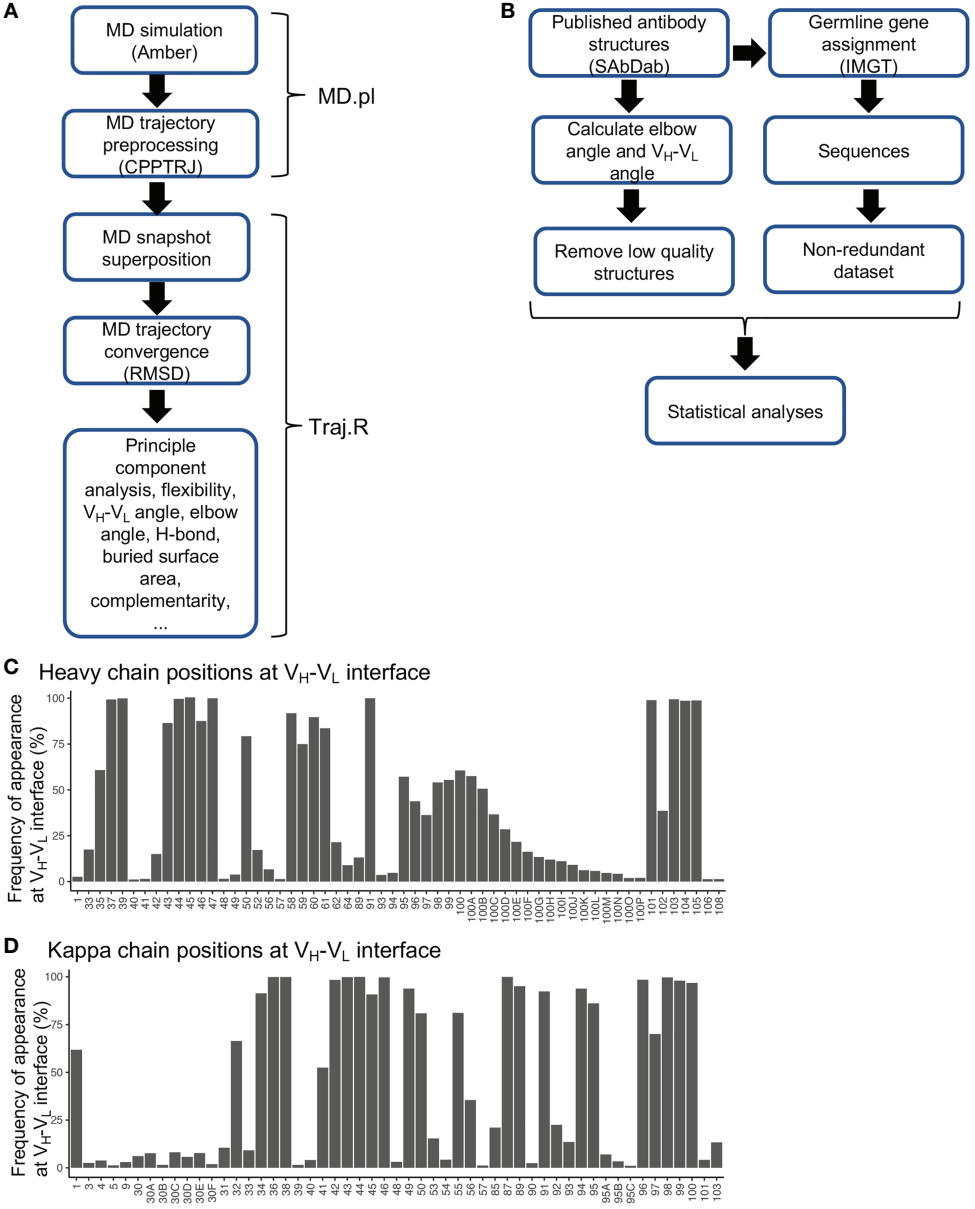
Diagrams for MD simulation and PDB structural analyses and frequencies of residues at V_H_-V_L_ interface. **(A)** Diagram of MD simulation and analyses performed. **(B)** Diagram for analyzing antibody structures from the PDB database. **(C)** Frequencies of heavy chain variable domain residue positions at V_H_-V_L_ interface. Residues with frequency less than 1% were omitted. **(D)** Frequencies of kappa chain variable domain residues at V_H_-V_L_ interface. Residues with frequency less than 1% were omitted.

To predict the effect of SHM on antibody conformation, we developed a script (MD.pl) to perform multiple steps of MD simulation from energy minimization, heating, equilibration, to production using Amber18 (Details see *Materials and Methods*) (**Figure 1A**). We also integrated multiple algorithms to develop a master script (Traj.R) to calculate structural features of antibodies from MD trajectory snapshots and to generate plots including root mean square deviation (RMSD), principle component analysis (PCA), V_H_-V_L_ angle (six parameters, **Figure S1A**), elbow angle, buried accessible surface area (bASA), dynamics of hydrogen bonds, and root mean square flexibility (RMSF) (**Figure 1A**).

Antibody structures determined at the residue-level contain rich information for exploring conformational alterations induced by SHMs. To better utilize this dataset, we collected a total of 5994 experimentally determined antibody structures from the SAbDab database (29). We removed redundant structures with identical heavy and light chain variable domain sequences to identify 3163 unique structures, with 2651 having the antigen-binding fragment (Fab). For each structure, antibody positions were assigned according to the Chothia numbering scheme. We annotated each structure with various features including gene origin, amino acid sequence, SHM level, V_H_-V_L_ angle, elbow angle, bASA at domain interfaces, and hydrogen bonds and salt bridges at domain interfaces (**Figure 1B** and **Table S1**). By utilizing this dataset, we identified residue positions frequently located at V_H_-V_L_ and elbow interfaces (**Figures 1C, D**, **S1B–E**). One interesting observation is that many residue positions at the framework 2 (FWR2) and FWR4 regions contribute to either V_H_-V_L_ or elbow interfaces (**Figures S1F–I**). In the gene-specific substitution profiles (GSSPs), which predict positional SHM preference, we found that many residue positions at the V_H_-V_L_ and elbow interfaces mutate with high frequencies and strong substitution preference (**Figures S1F–I**), implying that SHMs at these positions are frequently used to modulate V_H_-V_L_ and elbow conformations. However, for many of these positions, the effects of SHMs have not been elucidated.

### MD Simulation to Identify SHMs Modulating V_H_-V_L_ and Elbow Conformation of VRC01

**Figure 2:**
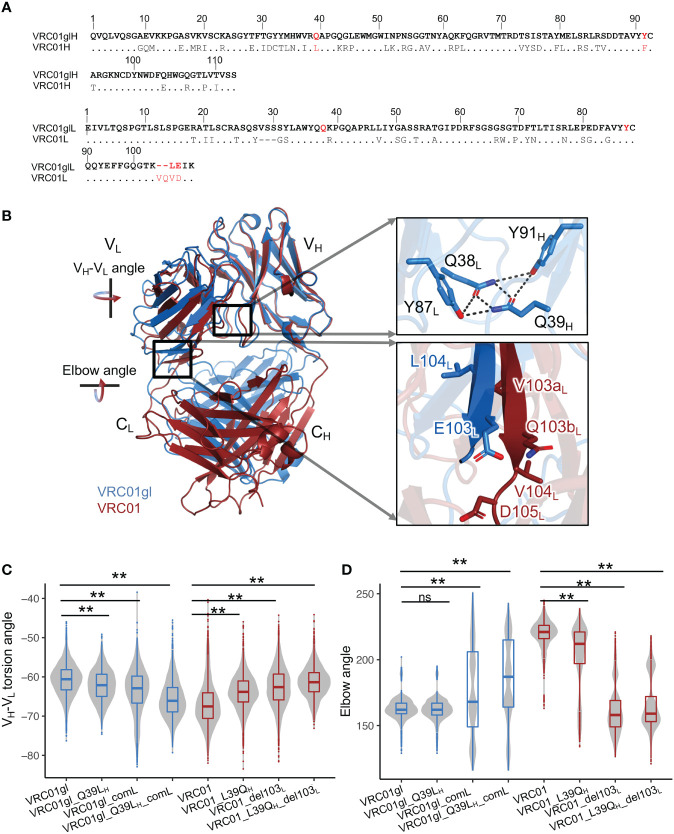
Somatic hypermutations involving Q39L_H_ and FWR4_L_ insertion modulate V_H_-V_L_ and elbow angles. **(A)** Heavy and light chain sequence alignments of VRC01gl and VRC01. VRC01 residues identical to VRC01gl are shown in dots. **(B)** Superimposition of structures of VRC01gl and VRC01 using heavy chain variable domain shows that V_H_-V_L_ and elbow conformation are changed in mature VRC01. **(C)** V_H_-V_L_ angle distribution of VRC01gl and VRC01 mutants from MD simulation. Other parameters for measuring the V_H_-V_L_ conformation are shown in **Figure S2C**. **(D)** Elbow angle distribution of VRC01gl and VRC01 mutants from MD simulation. Kolmogorov–Smirnov test is used to compare the significance of difference for V_H_-V_L_ and elbow angles. P values less than 0.01 are labeled with two stars. We use Bonferroni Correction to control false discover rate of multiple test <0.01. ns, not significant.

VRC01 is a broadly HIV-1 neutralizing antibody (bnAb) which is an important template for antibody-targeted vaccine design. Currently, immunogens (eOD-GT6, eOD-GT8, etc.) have been designed to activate the precursors of VRC01-like antibodies for affinity maturation (30, 31). VRC01 accumulates a high level of SHMs (**Figure 2A**). However, the effects of many SHMs in VRC01, which are also observed in numerous other antibodies, are unknown. The germline reverted VRC01 (VRC01gl) in complex with eOD-GT6 and VRC01 in complex with gp120 form a good model system to characterize the effects of SHMs through forward and reversion mutagenesis.

In particular, the comparisons of X-ray crystal structures of VRC01gl and VRC01 revealed dramatic differences in both V_H_-V_L_ and elbow angles (**Figure 2B**). To identify SHMs associated with the conformation changes, we introduced individual and combinations of a portion of SHMs observed at the V_H_-V_L_ and elbow interfaces in VRC01 to VRC01gl, and performed MD simulations accordingly. Our analyses showed that SHM-induced conformation changes in V_H_-V_L_ and elbow angles could be observed during long timescale (1μs) MD simulation (**Figures S2A, B**). Note, we referred to V_H_-V_L_ and elbow angle changes as changes in the sampled distributions or propensity during MD simulation. We used the torsion angle (HL), and four tilting angles (HC1, HC2, LC1, LC2), and one distance parameter defined by ABangle to quantify the V_H_-V_L_ angle (**Figure S1A**). We will mainly describe the V_H_-V_L_ torsion angle changes in the following analyses. MD simulation successfully identified critical SHM events associated with the VRC01 conformation change: heavy chain Q39L_H_, light chain ‘VQ’ insertion at 103_L_, L104V_L_, and E105D_L_ (**Figures 2A–D**, **S2C–H**). Q39_H_ is involved in a hydrogen bond (HB) network at the V_H_-V_L_ interface, which includes Q39_H_, Y91_H_, Q38_L_, and Y87_L_ (**Figure 2B**). This HB network is completely disrupted in VRC01 by two SHMs: Q39L_H_ and Y91F_H_ (**Figure 2A**). MD simulation revealed that Q39L_H_ increased the V_H_-V_L_ torsion angle of VRC01gl (**Figures 2C**, **S2C**). Consistently, the revertant L39Q_H_ in VRC01 decreased the V_H_-V_L_ torsion angle. However, substitutions at 39_H_ alone do not alter the elbow angles of VRC01 and VRC01gl (**Figure 2D**). Further analysis revealed that the combination of ‘VQ’ insertion at 103a_L_ and 103b_L_, L104V_L_, and E105D_L_ (the combination defined as comL) at light chain FWR4 is critical for the increased elbow angle in VRC01 (**Figure 2D**). The deletion of 103a_L_ and 103b_L_ (del103_L_) reduced the elbow angle and V_H_-V_L_ torsion angle of VRC01. Consistently, comL increased the V_H_-V_L_ torsion angle of VRC01gl and modulated VRC01gl to sample multiple elbow angle states including a state similar to VRC01, suggesting that the insertion increases the flexibility of the elbow interface. Moreover, only the combination of Q39L_H_ and comL but not individual mutations altered the V_H_-V_L_ angle of VRC01gl to a level comparable to VRC01 (**Figures S2C–H**). Consistently, the combination of del103_L_ and L39Q_H_ altered the V_H_-V_L_ and elbow angles of VRC01 to levels comparable to those of VRC01gl. Moreover, we observed that the combination of Q39L_H_ and comL increased the flexibility of heavy chain CDRs of VRC01gl, while the combination of L39Q_H_ and del103_L_ reduced the flexibility of VRC01 CDRs (**Figures S2I, J**). In summary, the forward mutations in VRC01gl and reversion mutations in VRC01 consistently revealed the SHMs modulating V_H_-V_L_ and elbow angles, with an additive effect between Q39L_H_ and comL.

### Effects of 39_H_ and FWR4_L_ SHMs on Binding Affinity

**Figure 3:**
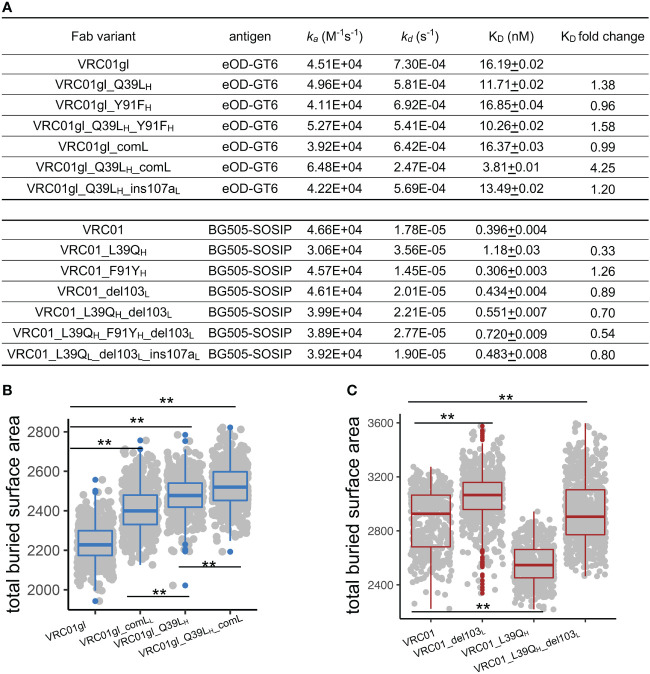
FWR somatic hypermutations improve antigen-binding affinity by increasing bASA between antibody and antigen. **(A)** Antigen-binding affinity of VRC01gl and VRC01 mutants measured by SPR. Q39L_H_ and comL improves VRC01gl binding affinity against eOD-GT6. L39Q_H_ revertant reduces binding affinity of VRC01 against BG505-SOSIP. **(B)** Q39L_H_ and comL increase bASA between VRC01gl and eOD-GT6. **(C)** L39Q_H_ and VRC01_del103_L_ reduce bASA between VRC01 and gp120. Kolmogorov–Smirnov test is used to compare the significance of difference. P values less than 0.01 are labeled with two stars.

To further explore the effects of 39_H_ and FWR4_L_ SHMs individually and in combination, we produced forward mutants of VRC01gl (Q39L_H_ and comL) and revertants of VRC01 (L39Q_H_ and del103_L_) and measured antigen-binding affinity, thermostability, and protein size. We measured K_D_s of VRC01gl and VRC01 variants against eOD-GT6 and BG505-DSSOSIP respectively using surface plasmon resonance (SPR). We used different antigens for VRC01gl and VRC01 because VRC01gl does not bind HIV-1 gp120/gp41 trimer. The SPR results revealed that Q39L_H_ or comL alone had a mild effect on VRC01gl K_D_, but the combination improved K_D_ by 4-fold (**Figures 3A**, **S3A**), primarily due to a slower reduced dissociation rate (*k_d_*). For VRC01, the revertant mutation L39Q_H_ decreased the K_D_ of VRC01 by 3-fold due to faster *k_d_*, while del103_L_ and the combination of L39Q_H_ and del103_L_ showed moderate decrease on the K_D_ (**Figure 3A**).

To understand the mechanism of binding affinity alteration by Q39L_H_ and comL, we performed MD simulations of VRC01gl/eOD-GT6 and VRC01/gp120 with and without the mutations. We found that each of the VRC01gl and VRC01 variants sampled V_H_-V_L_ torsion angle and elbow angle comparable to those observed in antigen-free MD simulations (**Figures S3B-D**), suggesting that the antibody variants undergo similar conformation changes in the presence of antigens. Both Q39L_H_ and comL increased the bASA between VRC01gl and eOD-GT6 over 200 Å², with the combination increasing the bASA the most (**Figure 3B**), consistent with the additive effect of the combination on V_H_-V_L_ and elbow conformation change. For the three VRC01 reverteants, L39Q_H_ decreased the bASA between VRC01 and gp120 (**Figure 3C**) the most, which is consistent with the SPR results. Above all, these results suggested that modulation of bASA between epitope and paratope, which may alter *k_d_*, is one mechanism for improving K_D_ by Q39L_H_ and comL

### Epistasis Between 39_H_ and FWR4_L_ SHMs on Stability and Aggregation

**Figure 4:**
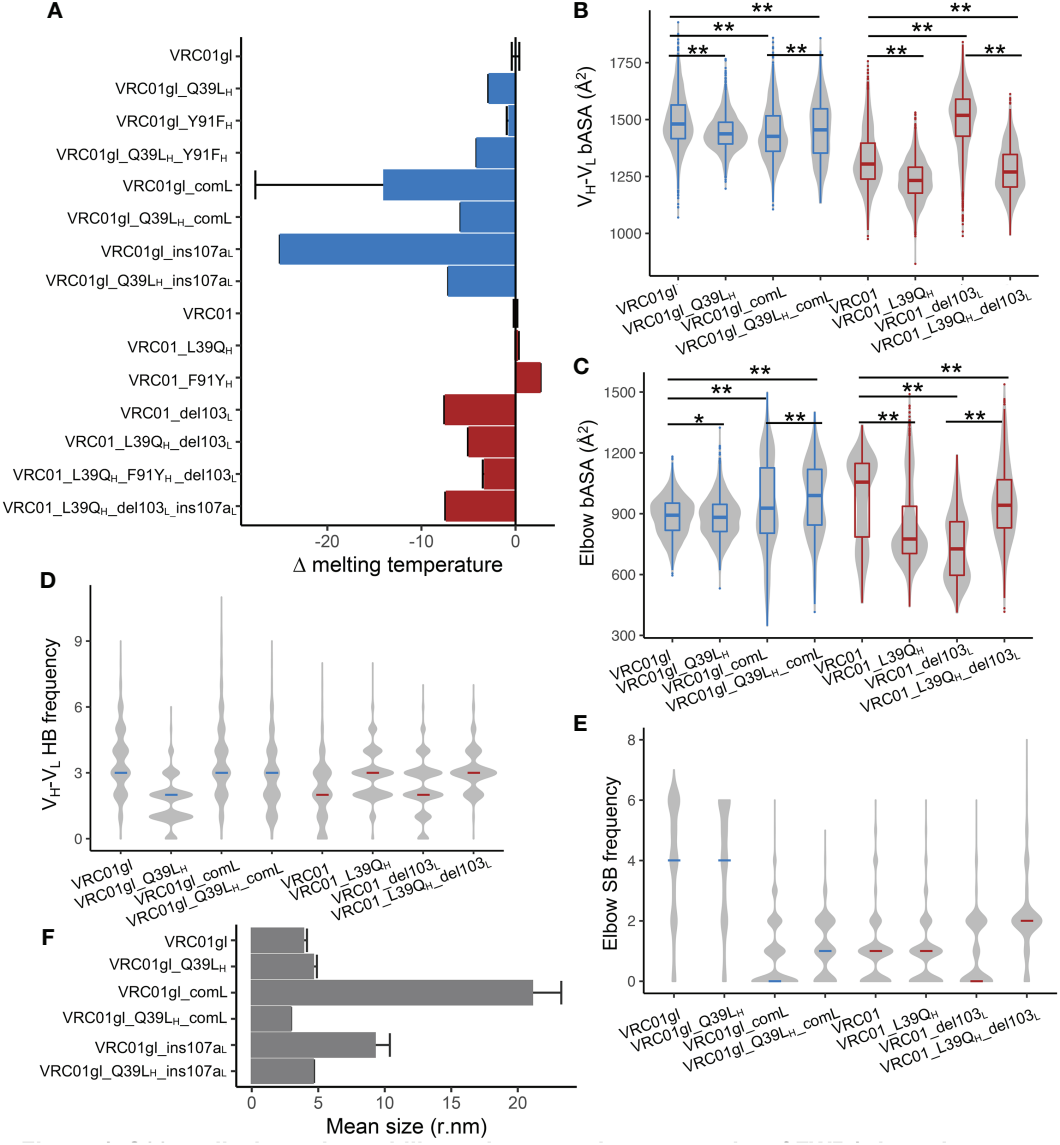
Q39L_H_ alleviates the stability and aggregation propensity of FWR4_L_ insertion. **(A)** Effects of somatic hypermutations on VRC01gl and VRC01 melting temperature. Data are shown with mean and SD from two replicates. **(B)** Buried accessible surface area between V_H_ and V_L_ domains obtained from MD simulation. MD repeats were combined for each antibody variant. **(C)** Buried accessible surface area between variable and constant domains obtained from MD simulation. MD repeats were combined for each antibody variant. **(D)** Frequencies of hydrogen bonds at V_H_-V_L_ interface in MD simulation. Median was highlighted by line. **(E)** Frequencies of salt bridges at elbow interface in MD simulation. Median was highlighted by line. **(F)** Effects of somatic hypermutations on VRC01gl and VRC01 Fab protein size. Data are shown with mean and SD from three replicates. Kolmogorov–Smirnov test is used to compare the significance of difference for panels **(B, C)**. P values less than 0.01 are labeled with two stars. P values greater than 0.01 and less than 0.05 are labeled with one star.

We next measured melting temperature (T_m_) to investigate the effects of 39_H_ and FWR4_L_ mutations on antibody stability. For VRC01gl, Q39L_H_ and comL destabilized VRC01gl (Δ T_m_ -2.8°C and -14.0°C respectively, **Figure 4A**). Surprisingly, the destabilization effect of comL was alleviated by Q39L_H_. For VRC01, the reversion of L93Q_H_ had a minor effect on stability (Δ T_m_ 0.1°C), while del103_L_ destabilized VRC01 by 7.4 °C. Interestingly, the destabilization effect of del103_L_ was attenuated by L39Q_H_.

Because the above mutations altered antibody conformation, we hypothesized that the observed stability changes may result from conformation change induced alterations in bASA and polar interactions in the V_H_-V_L_ and elbow interfaces. To examine this hypothesis, we calculated bASA of the V_H_-V_L_ and elbow interfaces as well as numbers of hydrogen bonds and salt bridges (SBs) in MD trajectories of each antibody variant. Note, stable SBs were not observed in the V_H_-V_L_ interface and thus excluded from the analysis. For VRC01gl, we found that Q39L_H_ reduced bASA of both V_H_-V_L_ and elbow interfaces (**Figures 4B, C**). Q39L_H_ also reduced the number of V_H_-V_L_ HBs mainly due to the disruption of the HBs between Q39_H_ and Q38_L_ (**Figures 4D**, **S4A**). All these changes together may account for the observed destabilization effect of Q39L_H_. ComL showed reduced bASA of the V_H_-V_L_ interface, increased bASA of the elbow interface, and reduced SBs at the elbow interface (**Figures 4B–E**). Interestingly, compared to comL alone, the combination of comL and Q39L_H_ showed increased bASA at both V_H_-V_L_ and elbow interfaces and SBs at elbow interface, consistent with the T_m_ measurement that Q39L_H_ improved the stability of the comL mutant. For VRC01, L39Q_H_ reduced bASA of both interfaces which may counteract the effect of increased HBs at the V_H_-V_L_ interface on stability (**Figures 4B–D**). The reduced bASA of elbow interface and number of SBs may be associated with the destabilization effect of del103_L_ (**Figures 4C, E**). In contrast, the addition of L39Q_H_ recovered the bASA and the number of SB decreases in the del103_L_ variant, coincided with the stability improvement (**Figure 4A**).

In addition, we noticed that VRC01gl with comL tended to precipitate, but the combination of comL and Q39L_H_ did not. We suspected that comL could result in VRC01gl aggregation and therefore used dynamic light scattering (DLS) to measure the sizes of VRC01gl variants. The DLS results showed that VRC01gl with comL was about 4-fold larger than the wildtype (**Figures 4F**, **S4C**), confirming that comL led to aggregation in VRC01gl. Notably, such aggregation disappeared when comL was combined with Q39L_H_.

In addition, we also assessed the effect of Y91F_H_, another residue involved in the HB network between V_H_ and V_L_. We found that Y91F_H_ and its reversion had mild effects on V_H_-V_L_ and elbow angles and binding affinities of VRC01gl and VRC01 variants (**Figures 3A**, **S2C–H**). Nonetheless, Y91F_H_ and Q39L_H_ and their reversions had an additive effect on VRC01gl and VRC01 stability (**Figure 4A**).

### Conservation, SHM Frequency, and Commonality of the Effects of 39_H_ and FWR4_L_ Insertion

**Figure 5:**
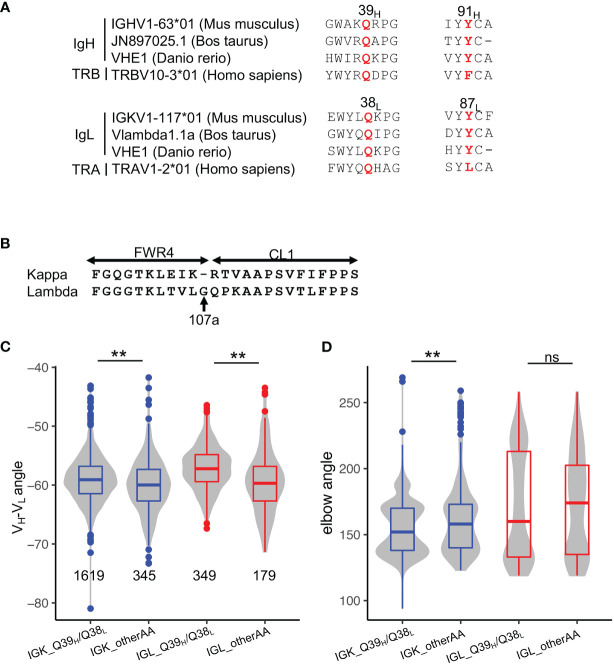
Disruption of the Q39_H_/Q38_L_ hydrogen bonds represents a common mechanism for altering V_H_-V_L_ conformation in affinity maturation. **(A)** Sequence alignment of Q39_H_, Y91_H_, Q38_L_, Y87_L_ in antibody V genes of three species and T-cell receptor V genes of humans. **(B)** Sequence alignment of kappa and lambda FWR4 and constant regions. **(C)** V_H_-V_L_ angles in PDB structures stratified by light chain isotype. Significance of the Kolmogorov–Smirnov test are shown on top. **(D)** Elbow angles in PDB structures stratified by light chain isotype. Significance of the Kolmogorov–Smirnov test are shown on top. P values less than 0.01 are labeled with two stars. ns, not significant.

To understand whether SHMs at 39_H_ and comL affect other antibodies, we first examined the conservation of all positions in germline genes. We observed Q39_H_, Y91_H_, Q38_L_, and Y87_L_ to be conserved in many germline V genes of both BCR and T cell receptors across species (**Figures 5A**, **S5A**), suggesting a common mechanism for stabilizing interdomain interactions of the immunoglobulin superfamily. Our search of the cAb-Rep database revealed 39_H_ to have a mutation frequency of approximately 4%, with Q39L_H_ the most prevalent (**Figure S1F**).

To examine the commonality of the effect of Q39_H_/Q38_L_ pairs on antibody conformation, we compared the V_H_-V_L_ and elbow angles of antibodies with and without the residue pair. Because lambda light chain is one residue longer than kappa chain (**Figure 5B**, named 107a_L_ according to kappa FWR4), resulting in increased elbow angle flexibility (32), we compared kappa and lambda chain antibodies separately. Overall, we found that both kappa and lambda antibodies with the Q39_H_/Q38_L_ pair have a lower V_H_-V_L_ torsion angle, consistent with the observation in VRC01gl (**Figures 5C, D**). However, the HC1, LC1, and LC2 angles of the two isotypes change in different directions (**Figures S5B–F**), with kappa antibodies showing directions of angle changes consistent with VRC01gl. Both kappa and lambda antibodies with the Q39_H_/Q38_L_ pair tend to have lower elbow angles. In summary, the analysis indicated that SHMs at 39_H_ and 38_L_ broadly modulate conformations of many antibodies, with the conformation change genetic-context dependent.

No insertion is observed in light chain germline J genes. The search of antibody transcripts in the cAb-Rep database revealed a frequency of insertion less than 10^-8^ summed over all FWR4_L_ positions, suggesting that comL is a rare event that is only observed in the VRC01 lineage. L104V_L_ and E105D_L_ are common individually (**Figure S1I**). To further examine whether the epistasis effect between 39_H_ and comL is confined to the ‘VQ’ insertion at 103_L_, we introduced G107a_L_, frequently observed in lambda chain FWR4, to VRC01gl as well as VRC01 with del103_L_. We found that G107aL significantly destabilized VRC01gl and formed aggregation, but was further stabilized by Q39L_H_ (**Figures 4A, F**, **S4C**). VRC01 with both del103_L_ and G107a_L_ could not be produced, but was expressed when combined with L39Q_H_. Furthermore, G107aL did not affect the binding affinity of VRC01gl and VRC01 variants (**Figure 3A**). In summary, the results demonstrated again the remote interaction between V_H_-V_L_ interface SHM and FWR4_L_ insertions.

### The Frequencies of SHMs at 39_H_ and 38_L_ Are High in HIV-1 nAbs

**Figure 6:**
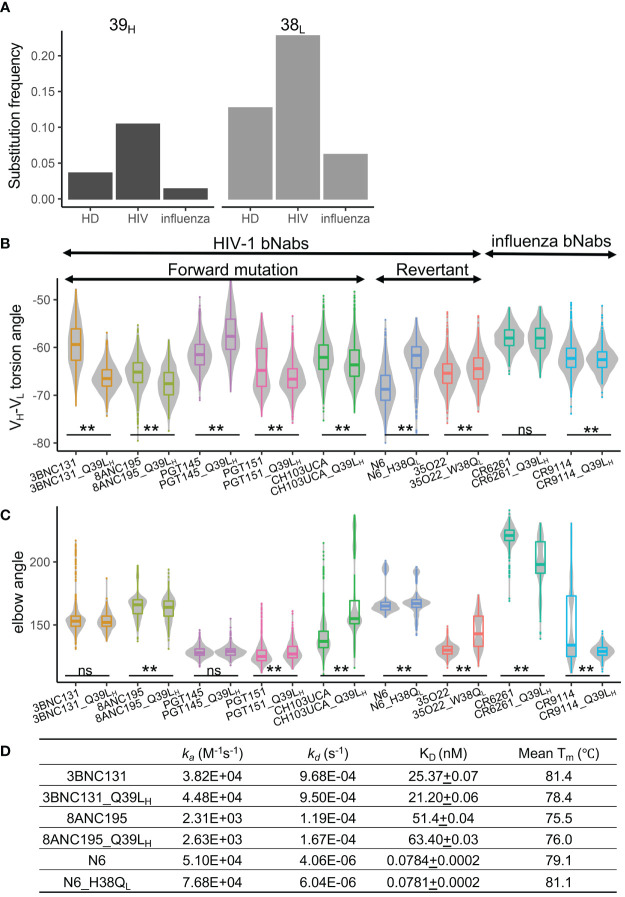
Somatic hypermutations at 39_H_ and 38_L_ are enriched in anti-HIV-1 antibodies. **(A)** Frequencies of somatic hypermutations at 39_H_ and 38_L_ increase in anti-HIV-1 antibodies. HD, healthy donor antibody repertoire. **(B)** Somatic hypermutations at 39_H_ and 38_L_ modulate the V_H_-V_L_ conformation of bnAbs. **(C)** Somatic hypermutations at 39_H_ and 38_L_ modulate the elbow conformation of HIV-1 and influenza bnAbs. P values less than 0.01 are labeled with two stars. ns, not significant. We use Bonferroni Correction to control false discover rate of multiple test <0.01. **(D)** Somatic hypermutations at 39H and 38L do not affect the binding affinity of three HIV-1 bnAbs.

To further understand the commonality of the effects of 39_H_ and 38_L_ SHMs, we analyzed the frequencies of 39_H_ and 38_L_ SHMs in anti-HIV-1 neutralizing antibodies and anti-influenza antibodies. We found that the SHM frequencies of 39_H_ and 38_L_ are higher in anti-HIV-1 antibodies than in the general antibody repertoire and anti-influenza antibodies (**Figures 6A**, **S6**).

To understand whether the conformational changes of 39_H_ and 38_L_ SHMs can be observed in other HIV-1 and influenza antibodies, we introduced or reverted 39_H_ and 38_L_ substitutions in six selected HIV-1 bnAbs, one HIV-1 bnAb unmutated common ancestor (UCA), and two influenza bnAbs and performed MD simulations. The results revealed that the V_H_-V_L_ angles of eight antibodies were affected by 39_H_ or 38_L_ mutations (**Figures 6B, C**). The direction of V_H_-V_L_ torsion angle changes in the six HIV-1 bnAbs is consistent with that in VRC01gl, except PGT145. For five anti-HIV-1 antibodies and both anti-influenza antibodies, the elbow angles were also affected (**Figure 6C**).

We then selected three anti-HIV-1 bnAbs (3BNC131, 8ANC195, and N6) and measured their binding affinity against BG505-SOSIP and thermostability. We found that the three antibodies with 39_H_ or 38_L_ mutations showed no difference in K_D_ compared to their respective wildtypes (**Figure 6D**). The stability changes in 3BNC131 and N6 were consistent with those observed in VRC01gl and VRC01. However, Q39L_H_ did not impair the stability of 8ANC195, suggesting that the effect of Q39L_H_ on stability is also context-dependent.

## Discussion

In this study, we established a structural bioinformatics pipeline to investigate the structural basis of the effects of somatic hypermutation. The pipeline was found to be successful at detecting SHM-induced conformational changes, with large conformation changes observed in MD simulation at a long timescale (μs). These conformation changes can be used to understand alterations in binding affinity, stability, flexibility, and other antibody features. With the development of high-performance GPUs, the pipeline paved the way for elucidating mechanisms of SHM effects in a high-throughput way. By using this pipeline, we revealed structural mechanisms of V_H_-V_L_ and elbow conformation changes induced by SHMs at positions 39_H_, 91_H_, 38_L_, and 87_L_ as well as FWR4_L_ insertions. The stabilization of FWR4_L_ insertion by alteration of V_H_-V_L_ conformation suggested a remote synergy between V_H_-V_L_ and elbow conformations. We also found that adjustment of V_H_-V_L_ conformation is a strategy frequently used by anti-HIV-1 nAbs for affinity maturation.

In general, FWRs scaffold CDRs and many FWR positions are less tolerant of SHMs than CDR residues, which is one of the reasons that many FWR positions undergo strong purifying selection during affinity maturation (12). The GSSP profiles revealed that FWR positions close to V_H_-V_L_ and elbow interfaces accumulate less SHMs than CDRs and loops in FWRs. Nonetheless, FWR SHMs are required for affinity maturation of numerous antibodies (16). In this study, we characterized the effects of SHMs involved in an HB-network at the V_H_-V_L_ interface at positions 39_H_, 91_H_, 38_L_, and 87_L_. Although the importance of the HB network has been reported previously (33, 34), the structural basis, commonality, and genetic-context dependence of its roles have not been revealed. Through altering V_H_-V_L_ orientation, SHMs at the four positions modulate multiple antibody features including binding affinity, stability, flexibility, and aggregation propensity. For individual antibodies, SHMs may be accumulated at these positions for some but not all of these effects. For example, Q38V_L_ increases V_H_-V_L_ torsion angle which reduces the steric clash between gp120 and anti-HIV-1 antibody CH103 UCA; Q39L_H_ also improved the binding affinity of CH103 UCA, but the structural basis was not explored (34). In the current study, we found that CH103 UCA with Q39L_H_ contained both V_H_-V_L_ and elbow orientations more similar to those of matured CH103 characterized in a previous study (19), which could account for the binding affinity improvement. The current study also uncovered that the V_H_-V_L_ orientation change induced by 39_H_ can improve binding affinity by reducing the dissociation constant *k_d_* through increasing bASA between antibody and antigen. As a side effect, individual SHMs at 39_H_, 91_H_, 38_L_, and 87_L_ destabilized antibodies by 1-3 °C. Furthermore, despite 39_H_ and 38_L_ forward and reverse mutations altered V_H_-V_L_ orientation of anti-HIV-1 bnAbs (**Figure 6B**), we did not observe changes in binding affinity. It is possible that these SHMs cooperated with other SHMs for function similar to the observations in VRC01gl. Another possibility is that bnAbs may accumulate SHMs at 39_H_ and 38_L_ for stability or other beneficial effects at certain stages of the affinity maturation process. As more SHMs accumulate, the effects of individual 39_H_ and 38_L_ mutations may be counteracted in the matured bnAbs. Thus, antibodies with low or no SHM will be ideal for investigating the effect of individual SHMs. We acknowledge that the MD simulation predicted conformation changes induced by 39_H_ and 38_L_ SHMs were not validated by experimental approaches in this study, but the consistency between the predictions and the PDB dataset analysis suggests that the predictions are reliable.

The dynamics of V_H_-V_L_ orientation tend to be interconnected with elbow orientation. Two previous studies revealed that mutations at the elbow interface increase V_H_-V_L_ torsion angle through increasing elbow angle (19, 20). The current study showed that V_H_-V_L_ interface SHMs can also increase elbow angle. Moreover, as kappa chains are one residue shorter in FWR4_L_, they tend to have a larger energy barrier to adopting elbow orientations with angles greater than 200 degrees than lambda chain (**Figure 4**). Insertions in FWR4_L_ reduced the energy barrier and increased the flexibility of the elbow region, but resulted in destabilization and aggregation of VRC01gl. Nonetheless, we observed that introducing Q39L_H_ alleviated the aggregation and improved the stability of VRC01gl, possibly because the V_H_-V_L_ HB network rigidifies the V_H_-V_L_ conformation to be incompatible with the large elbow angle. Surprisingly, we also observed that the VRC01 G107aL variant can only be produced in the presence of L39Q_H_, which alters VRC01 antibody conformation. Thus, the alteration of V_H_-V_L_ and elbow conformations may represent a general way to reduce aggregation propensity. Because the DLS results showed a single peak in the size measurement for VRC01gl with comL and G107a_L_, implying that the two variants form a stable Fab tetramer and dimer respectively. Although the structures of the polymers are unclear, they may not affect the SPR readout because the antibody variants were captured on the SPR chip surface through their constant domain and the paratopes are probably available for antigen-binding. Furthermore, the destabilization effect of FWR4_L_ insertions suggested that its occurrence is limited to certain V_H_-V_L_ conformation contexts, which may explain the rarity of indels in FWR4 regions of both heavy and light chains. Above all, there is a remote epistasis between mutations in the V_H_-V_L_ and FWR4_L_ interfaces. However, further study is required to address whether the destabilization effect is VRC01gl specific.

V_H_-V_L_ orientation is essential for maintaining bioactivity when grafting CDRs of animal origins to the human FWR backbone (35, 36). We believe the pipeline established in this study provides an approach to predict V_H_-V_L_ orientations. The combination of SHMs at 39_H_, 91_H_, 38_L_, and 87_L_ and FWR4_L_ insertion provides a new way to alter antibody conformation. This study also highlighted the importance of genetic context for interrogating the effects of SHMs in antibody affinity maturation. In addition, some antibodies require reduced V_H_-V_L_ torsion angle and elbow angle for affinity improvement (20), a thorough understanding of the roles of many FWR SHMs, especially frequent SHMs, will help to generate a ‘dictionary’ for knowledge-based antibody engineering in different settings.

## Materials and Methods

### Molecular Dynamics Simulation

Antibody Fab structures for MD simulation were downloaded from PDB database (PDB IDs: VRC01gl, 4JPK; VRC01, 3NGB; CH103 UCA, 4QHK; PGT145, 3U1S; PGT151, 4NUG; 35O22, 4TOY; 3BNC131, 4RWY; 8ANC195, 4P9M; N6, 5TE7; CR9114, 4FQH; CR6261, 5C0S). We used FoldX (37) to introduce SHMs to antibody Fab structures. Modeller v9.16 with default parameters was used to model antibodies with insertion or deletion (38). For each Fab variant, we used tleap program to add a 10 angstrom (Å) cubic water box to the system, to neutralize the charge, and to generate topology and parameter files for MD simulation. For MD simulations of VRC01gl/eOD-GT6 and VRC01/gp120 complexes, to mimic the effects of N-glycosylation on antibody/antigen complexes, we built a MAN3 structure in tleap and replaced the crystal structure NAGs with MAN3 at N-glycosylation sites of eOD-GT6 and gp120 in PyMOL (39).

Amber18 with the amber14 and GLYCAM_06j-1 force fields was used for MD simulation (40, 41). We performed 1μs isothermal isobaric MD simulation per run (after 10,000 steps of solution energy minimization, 10,000 steps of whole system energy minimization, 5ns for heating from 0k to 300k, and 10ns of equilibration in the isothermal isovolumetric ensemble) on each Fab variant. 2-5 MD simulation repeats per Fab variant were performed with repeats not converged removed. A master MD script (MD.pl) was written to perform the above steps of MD simulation including energy minimization, heating, NPT and NVT ensembles.

### MD Trajectory Analysis

Bio3D in R was used to perform most of the trajectory analyses. For each MD run, snapshots were superimposed to the first snapshot using Cα atoms of the heavy and light chain variable domains and root-mean-square deviation (RMSD) was calculated to determine simulation convergence (42). After confirming convergence, snapshots of about 500ns were used for analyzing antibody conformational features. Root-Mean-Square Fluctuations (RMSF) were then calculated for each variable domain residue to examine whether SHMs modulate antibody flexibility. For each snapshot, we quantified the sampled distributions of the torsion and tilting angles and distance between V_H_ and V_L_ using ABangle recompiled in R (43), elbow angle by PyMOL, buried ASA and HB networks between domain interfaces using PISA (44). All statistical analyses in this study were performed in R. A master analysis script (Traj.R) was used to perform the above analyses.

### A Non-Redundant Antibody Structure Dataset

Experimentally determined antibody structures were downloaded from the SAbPred database (29). Gene origin and somatic hypermutation levels of each antibody were retrieved from the IMGT database (45). The elbow angle was calculated in PyMOL. bASA, hydrogen bonds at domain interfaces, and V_H_-V_L_ and elbow interface residues were calculated using PISA. We removed antibodies with identical heavy and light chain variable domain sequences using USEARCH (46) to obtain a non-redundant dataset.

### Gene-Specific Substitution Profiles (GSSPs) and Frequency of J Region Indel

The GSSPs for V genes were obtained from the cAb-Rep database (11). We used the mGSSP program to generate GSSPs as well as to identify insertions and deletions for all human J genes using all curated human antibody transcripts (~306 million) in the cAb-Rep database (12).

### Plasmid Design and Cloning

Genes encoding for the heavy (V_H_ & C_H_1 domains) and light (Kappa or Lambda V_L_ & C_L_ domains) chains of antibody Fabs were cloned in the mammalian expression plasmid pVRC8400. eOD-GT6 was cloned into the mammalian expression plasmid pHL-sec between the AgeI and KpnI sites. VRC01 heavy chain constructs were followed by a C-terminal octa-histidine tag and eOD-GT6 a hexa-histidine tag. The VRC01 light chain constructs had no tags.

### Site-Directed Mutagenesis Using Double-Primer PCR

Fabs mutants were generated using Pfu Ultra II polymerase in a protocol that employed both forward and reverse primers in the same PCR reaction for 18 cycles. The PCR products were denatured, and then reannealed. The non-mutated methylated parental plasmid was digested with DpnI and the remaining plasmids were transformed into E. coli cells. For each transformation, we selected five colonies at random and grew overnight in 5 ml LB + Kanamycin (pVRC8400) medium at 37°C. The plasmids were isolated using Spin miniprep kit (Qiagen, Germany) and sequenced to obtain the desired mutants.

### Protein Expression and Purification

Recombinant antibody Fabs were transiently expressed in FreeStyleTM 293F (Invitrogen) suspension cultures by co-transfection of pVRC8400 plasmids containing expression constructs for light chain and Fab heavy chain using polyethyleneimine (Polysciences). Cell growth was harvested on day 6 post transfection. eOD-GT6 was also produced in FreeStyleTM 293F (Invitrogen) suspension cultures by transient transfection using polyethyleneimine (Polysciences) of a pHLSec plasmid containing mammalian codon-optimized eOD-GT6 with a C-terminal Avi and His6x affinity tag. Proteins were harvested from the supernatant after 96 h.

The secreted proteins were purified by using Ni-NTA IMAC Sepharose 6 Fast Flow resin (GE Healthcare) nickel affinity chromatography followed by size exclusion chromatography (SEC) using a Superdex 200 26/600 (Fabs) or Superdex 75 26/600 (eOD) column (GE Healthcare) in 10 mM Tris pH 8.0, 150 mM NaCl SEC buffer. Peak fractions containing Fabs or eOD-GT6 were pooled. Protein purity was analyzed by SDS-PAGE and concentrated where possible to ~10 mg/mL. BG505-SOSIP was requested from the Vaccine Research Center at the National Institute of Health (47).

### Thermostability Measurements

Thermostability was measured by nano differential scanning fluorimetry on a Nanotemper Tycho NT. 6 instrument (NanoTemper Technologies) with a back-reflection aggregation detection at a range from 35 to 95°C and with a heating rate of 30°C/min. Protein unfolding was followed by tryptophan and tyrosine fluorescence intensity at 330 and 350 nm. The melting temperature (T_m_) was determined by detecting the maximum of the first derivative of the fluorescence ratios (F350/F330) after fitting experimental data with a polynomial function. Each sample was measured in duplicate or triplicate.

### Surface Plasmon Resonance (SPR)

SPR binding assays were performed using a Biacore T200 biosensor, equipped with a Series S CM5 chip, at 25°C in a HBS buffer, pH 7.4 (10mM HEPES pH 7.4, 150mM NaCl) supplemented with 0.1 mg/mL BSA and 0.005% (v/v) Tween-20.

For experiments involving the eOD-GT6 antigen, Fabs were captured to the chip surface using a human anti-Fab antibody (Human Fab Capture Kit, Cytiva), which was immobilized over all four flow cells of a chip using amine-coupling chemistry. Three different Fabs were captured over independent flow cells at 5-10 μg/mL at a capture level of approximately 400 RU. A surface without captured Fab served as a reference control. eOD-GT6 antigen was prepared in running buffer using a three-fold dilution series at six concentrations ranging from 2-486 nM, which were injected over all four flow cells simultaneously to increase concentration, using a 150s association time and 600s dissociation time at 50μL/min. At the end of each cycle the anti-Fab surface was regenerated using two consecutive 10s injections of 10 mM H_3_PO_4_ at 100μL/min, removing any Fab/eOD-GT6 bound complex, so that Fab can be re-captured in the next cycle. Buffer cycles instead of antigen samples were incorporated every two binding cycles to double reference the binding responses. Each concentration series was tested in triplicate.

BG505-SOSIP was tethered to the chip surface using the antibody 2G12 (NIH AIDS Reagent Program), which was immobilized over two flow cells of a Series S CM5 chip using amine-coupling chemistry. At the beginning of each cycle, BG505-SOSIP was captured over a single flow cell only, at 15ug/mL resulting in captures of approximately 400 RU, with the second flow cell used as a reference control. Fabs were used as the analyte at five concentrations ranging from 2.22-180 nM, with the exception of 8ANC195 and 8ANC195_Q39LH, which were tested at a 9-fold higher concentration range from 20-1620nM, to account for the slower association rate of the bound complex. Fab concentrations were prepared in running buffer using a three-fold dilution series and injected to increase concentration at 50μL/min for 150s association time and 900s dissociation time. 3BNC131 and 3BNC131_Q39LH, used dissociation times of 600s, and N6 and N6_Q39LH, used dissociation times of 2400s respectively to account for either a faster or a slower dissociating complex. The 2G12 surface was regenerated using a 10s pulse of 3M MgCl_2_ at 100μL/min, removing any bound BG505-SOSIP/Fab complex. Buffer cycles instead of antigen samples were incorporated every two binding cycles to double reference the binding responses. Each of concentration series was tested in triplicate.

Binding data were processed and fit to a simple 1:1 interaction model using the Scrubber 2.0 (BioLogic Software). The number in brackets reported with all kinetic parameters represents the error of the fit.

### Dynamic Light Scattering

The sizes of antibody Fabs purified with the nickel affinity chromatography were measured using Malvern Nano-ZS with a 173° detection angle at 20°C. Zetasizer v7.13 was used to calculate the size of each Fab.

## Data Availability Statement

The computational codes used in this study are available at https://github.com/shengzizhang/Antibody_MD.git. The anti-HIV-1 and anti-influenza antibodies were downloaded from the HIV molecular immunology database (http://www.hiv.lanl.gov/content/immunology) and RAPID database respectively (48). Antibody repertoire dataset was obtained from the cAb-Rep database (11).

## Author Contributions

ZS and LS designed the research. ZS wrote the MD simulation pipeline. ZS, YCG, YZG, and HZ analyzed data. JB and SP produced the antibody and antigens and performed melting temperature analysis. ZS and JB performed Dynamics Light Scattering analysis. PSK and LS performed SPR measurement. PDK provided reagents. LS and ZS wrote the paper. All authors reviewed, commented on, and approved the manuscript.

## Funding

Support for this work was provided by the National Institute of Allergy and Infectious Diseases (NIAID) grant R21 1R21AI138024-01A1 and the startup fund UR010655/70003/ZS2248 to ZS; support was also provided by the Intramural Research Program of the Vaccine Research Center, NIAID, NIH.

## Conflict of Interest

The authors declare that the research was conducted in the absence of any commercial or financial relationships that could be construed as a potential conflict of interest.

## Publisher’s Note

All claims expressed in this article are solely those of the authors and do not necessarily represent those of their affiliated organizations, or those of the publisher, the editors and the reviewers. Any product that may be evaluated in this article, or claim that may be made by its manufacturer, is not guaranteed or endorsed by the publisher.
