# Supplementary material for: Structural Basis of Antibody Conformation and Stability Modulation by Framework Somatic Hypermutation
Source: Front Immunol. 2022 Jan 3;12:811632. doi: 10.3389/fimmu.2021.811632 (PMC8761896; doi:10.3389/fimmu.2021.811632)
Supplement: Supplementary file 1 [file Presentation_1.pptx]

## Slide 1
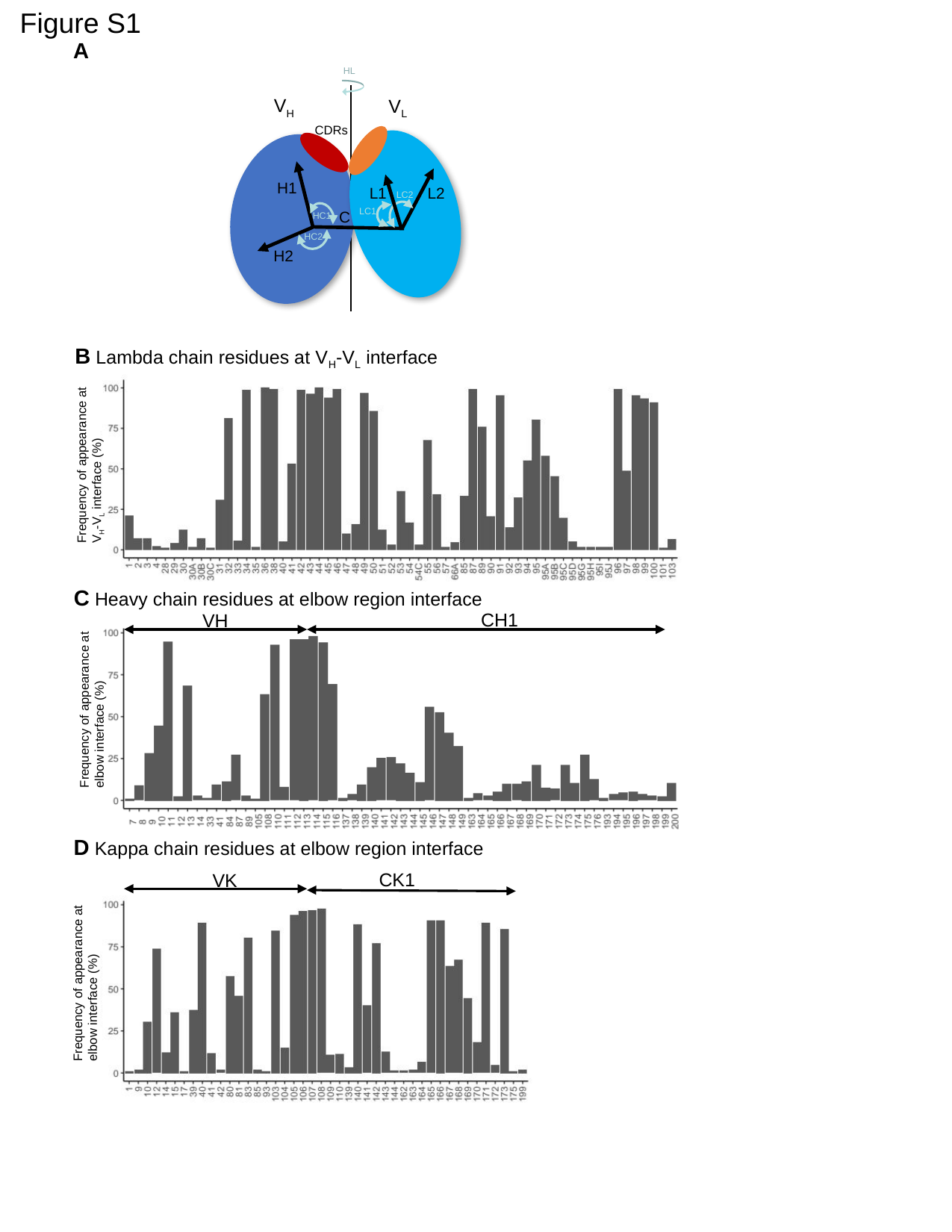

# Figure S1
A
HL
VH
VL
CDRs
H1
L1
L2
LC2
LC1
C
HC1
HC2
H2
B Lambda chain residues at VH-VL interface
Frequency of appearance at VH-VL interface (%)
C Heavy chain residues at elbow region interface
CH1
VH
Frequency of appearance at elbow interface (%)
D Kappa chain residues at elbow region interface
CK1
VK
Frequency of appearance at elbow interface (%)

## Slide 2
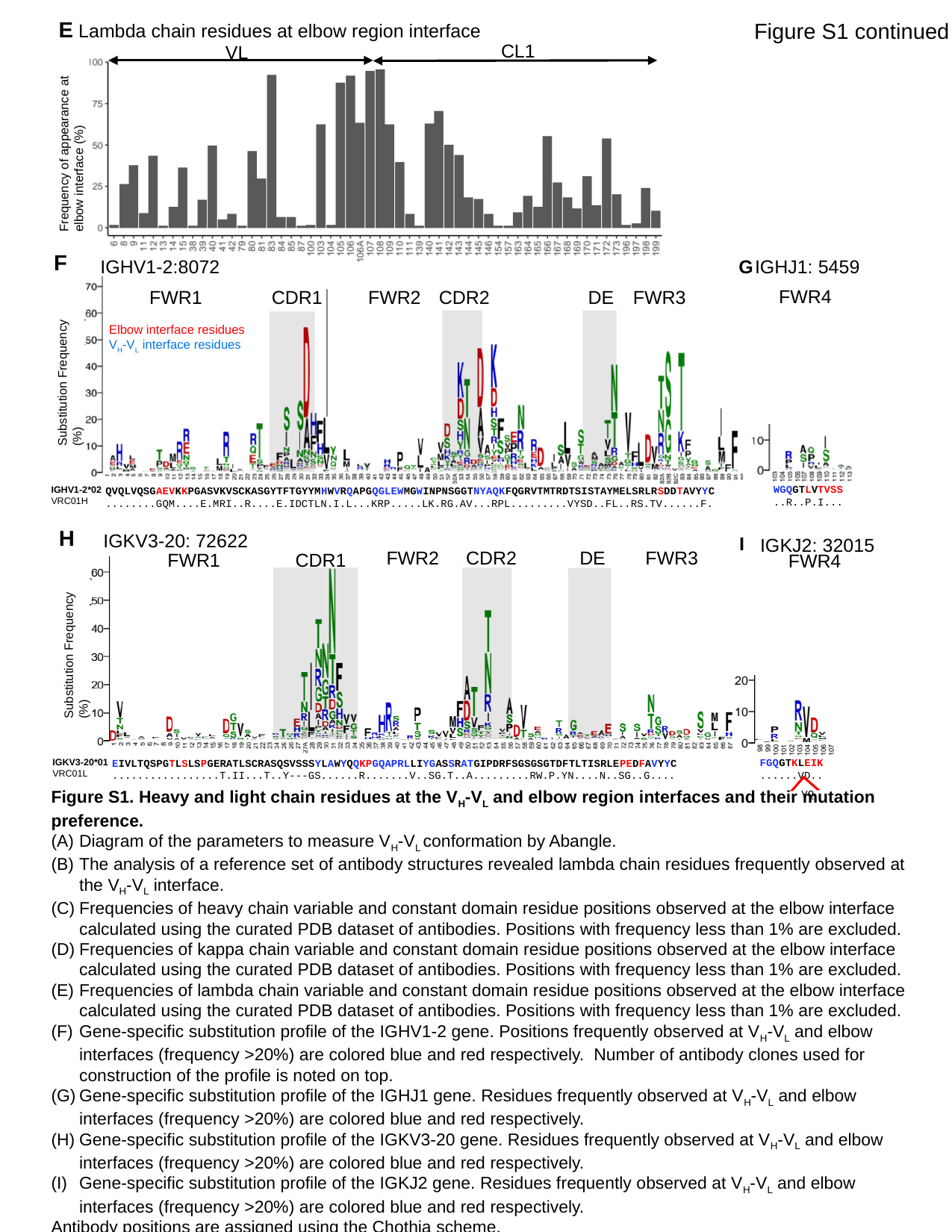

# Figure S1 continued
E Lambda chain residues at elbow region interface
CL1
VL
Frequency of appearance at elbow interface (%)
F
IGHV1-2:8072
G
IGHJ1: 5459
FWR4
FWR1
CDR1
FWR2
CDR2
DE
FWR3
Elbow interface residues
VH-VL interface residues
Substitution Frequency (%)
WGQGTLVTVSS
..R..P.I...
IGHV1-2*02
VRC01H
QVQLVQSGAEVKKPGASVKVSCKASGYTFTGYYMHWVRQAPGQGLEWMGWINPNSGGTNYAQKFQGRVTMTRDTSISTAYMELSRLRSDDTAVYYC
........GQM....E.MRI..R....E.IDCTLN.I.L...KRP.....LK.RG.AV...RPL.........VYSD..FL..RS.TV......F.
H
IGKV3-20: 72622
I
IGKJ2: 32015
FWR2
CDR2
DE
FWR3
CDR1
FWR1
FWR4
Substitution Frequency (%)
FGQGTKLEIK
......VD..
EIVLTQSPGTLSLSPGERATLSCRASQSVSSSYLAWYQQKPGQAPRLLIYGASSRATGIPDRFSGSGSGTDFTLTISRLEPEDFAVYYC
.................T.II...T..Y---GS......R.......V..SG.T..A.........RW.P.YN....N..SG..G....
IGKV3-20*01
VRC01L
Figure S1. Heavy and light chain residues at the VH-VL and elbow region interfaces and their mutation preference.
Diagram of the parameters to measure VH-VL conformation by Abangle.
The analysis of a reference set of antibody structures revealed lambda chain residues frequently observed at the VH-VL interface.
Frequencies of heavy chain variable and constant domain residue positions observed at the elbow interface calculated using the curated PDB dataset of antibodies. Positions with frequency less than 1% are excluded.
Frequencies of kappa chain variable and constant domain residue positions observed at the elbow interface calculated using the curated PDB dataset of antibodies. Positions with frequency less than 1% are excluded.
Frequencies of lambda chain variable and constant domain residue positions observed at the elbow interface calculated using the curated PDB dataset of antibodies. Positions with frequency less than 1% are excluded.
Gene-specific substitution profile of the IGHV1-2 gene. Positions frequently observed at VH-VL and elbow interfaces (frequency >20%) are colored blue and red respectively. Number of antibody clones used for construction of the profile is noted on top.
Gene-specific substitution profile of the IGHJ1 gene. Residues frequently observed at VH-VL and elbow interfaces (frequency >20%) are colored blue and red respectively.
Gene-specific substitution profile of the IGKV3-20 gene. Residues frequently observed at VH-VL and elbow interfaces (frequency >20%) are colored blue and red respectively.
Gene-specific substitution profile of the IGKJ2 gene. Residues frequently observed at VH-VL and elbow interfaces (frequency >20%) are colored blue and red respectively.
Antibody positions are assigned using the Chothia scheme.
VQ

## Slide 3
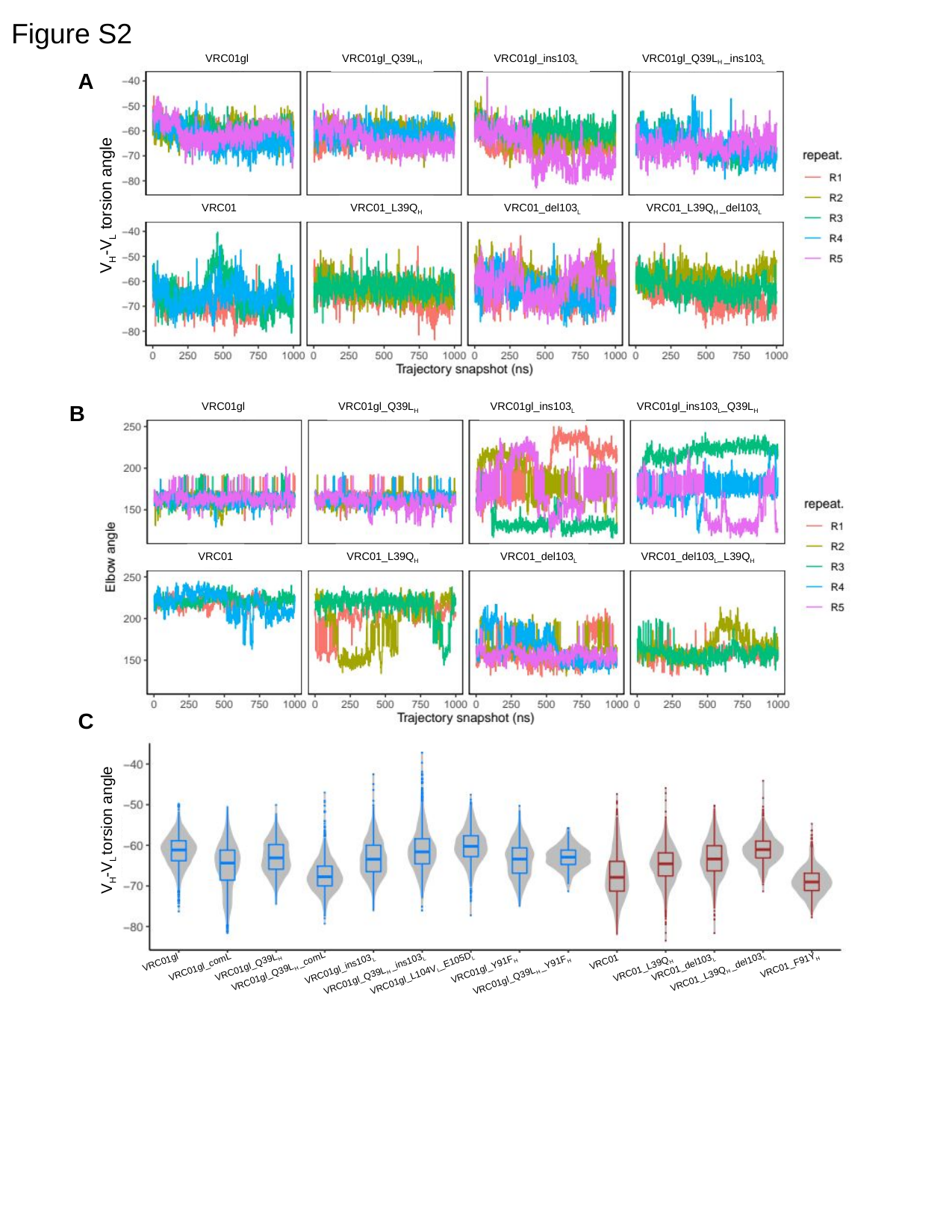

# Figure S2
VRC01gl
VRC01gl_Q39LH
VRC01gl_ins103L
VRC01gl_Q39LH _ins103L
A
VH-VL torsion angle
VRC01
VRC01_L39QH
VRC01_del103L
VRC01_L39QH _del103L
B
VRC01gl
VRC01gl_Q39LH
VRC01gl_ins103L
VRC01gl_ins103L_Q39LH
VRC01
VRC01_L39QH
VRC01_del103L
VRC01_del103L_L39QH
C
VH-VL torsion angle
VRC01
VRC01gl
VRC01_F91YH
VRC01gl_Q39LH
VRC01_del103L
VRC01gl_comL
VRC01_L39QH
VRC01gl_ins103L
VRC01gl_Y91FH
VRC01gl_Q39LH _comL
VRC01_L39QH _del103L
VRC01gl_L104VL_E105DL
VRC01gl_Q39LH _ins103L
VRC01gl_Q39LH _Y91FH

## Slide 4
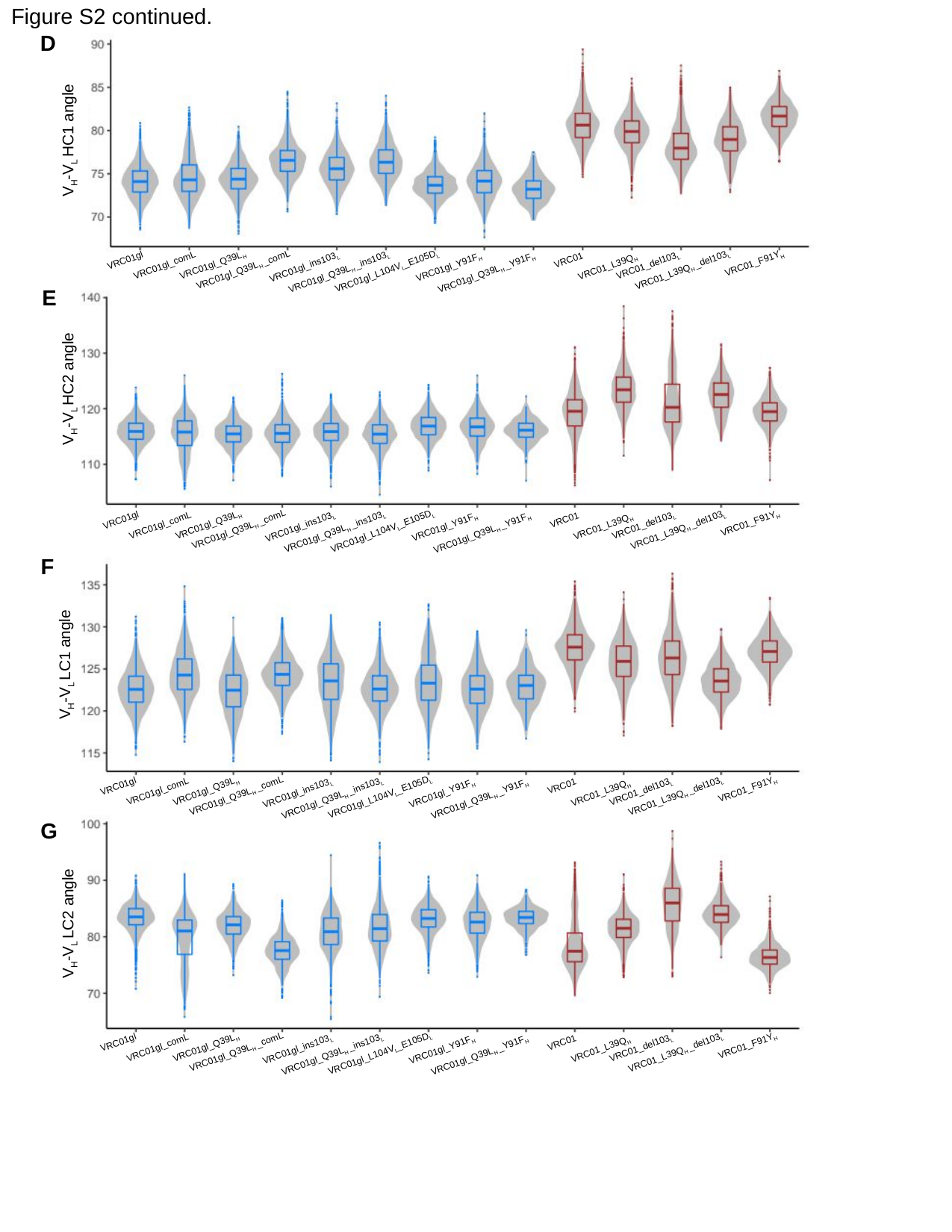

# Figure S2 continued.
D
VH-VL HC1 angle
VRC01
VRC01gl
VRC01_F91YH
VRC01gl_Q39LH
VRC01_del103L
VRC01gl_comL
VRC01_L39QH
VRC01gl_ins103L
VRC01gl_Y91FH
VRC01gl_Q39LH _comL
VRC01_L39QH _del103L
VRC01gl_L104VL_E105DL
VRC01gl_Q39LH _ins103L
VRC01gl_Q39LH _Y91FH
E
VH-VL HC2 angle
VRC01
VRC01gl
VRC01_F91YH
VRC01gl_Q39LH
VRC01_del103L
VRC01gl_comL
VRC01_L39QH
VRC01gl_ins103L
VRC01gl_Y91FH
VRC01gl_Q39LH _comL
VRC01_L39QH _del103L
VRC01gl_L104VL_E105DL
VRC01gl_Q39LH _ins103L
VRC01gl_Q39LH _Y91FH
F
VH-VL LC1 angle
VRC01
VRC01gl
VRC01_F91YH
VRC01gl_Q39LH
VRC01_del103L
VRC01gl_comL
VRC01_L39QH
VRC01gl_ins103L
VRC01gl_Y91FH
VRC01gl_Q39LH _comL
VRC01_L39QH _del103L
VRC01gl_L104VL_E105DL
VRC01gl_Q39LH _ins103L
VRC01gl_Q39LH _Y91FH
G
VH-VL LC2 angle
VRC01
VRC01gl
VRC01_F91YH
VRC01gl_Q39LH
VRC01_del103L
VRC01gl_comL
VRC01_L39QH
VRC01gl_ins103L
VRC01gl_Y91FH
VRC01gl_Q39LH _comL
VRC01_L39QH _del103L
VRC01gl_L104VL_E105DL
VRC01gl_Q39LH _ins103L
VRC01gl_Q39LH _Y91FH

## Slide 5
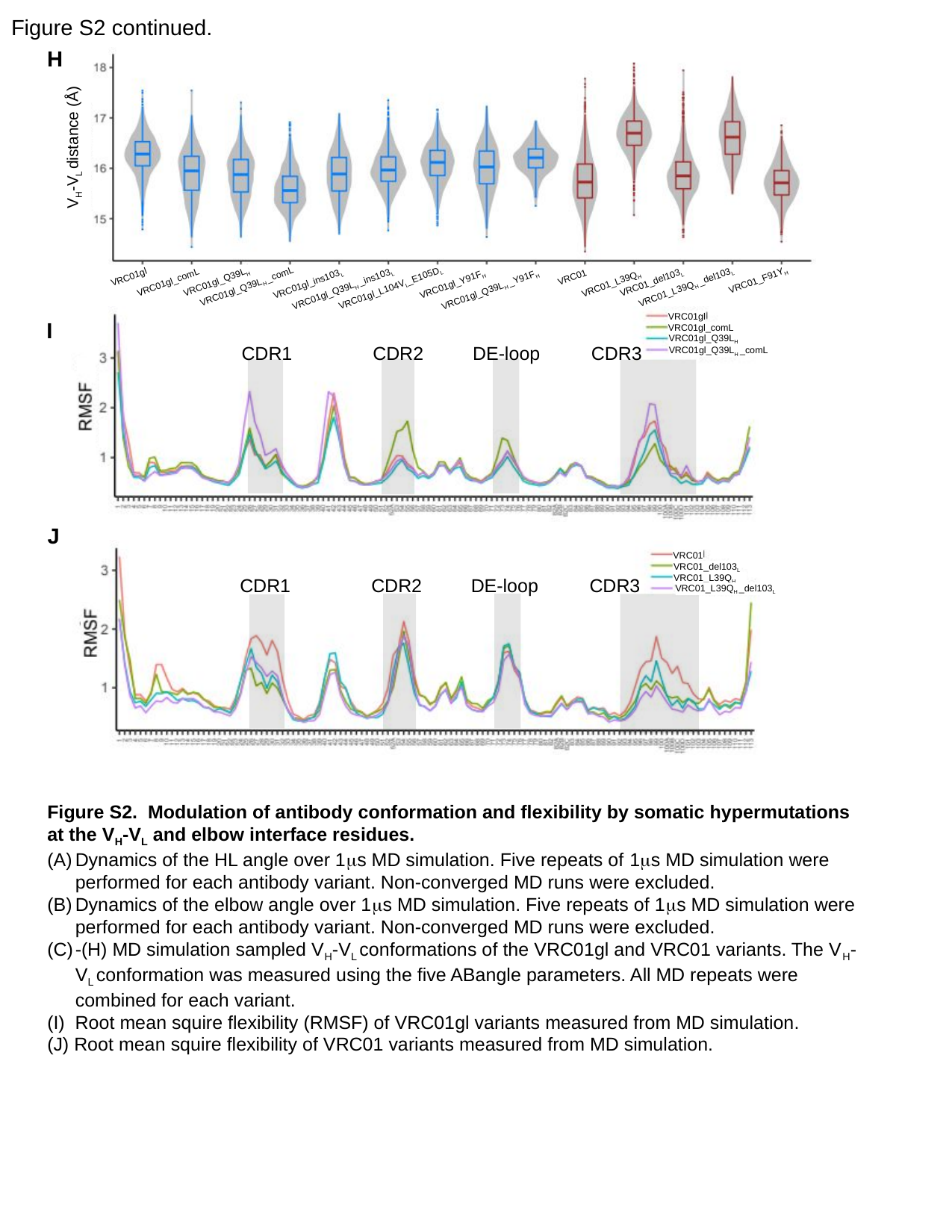

# Figure S2 continued.
H
VH-VL distance (Å)
VRC01
VRC01gl
VRC01_F91YH
VRC01gl_Q39LH
VRC01_del103L
VRC01gl_comL
VRC01_L39QH
VRC01gl_ins103L
VRC01gl_Y91FH
VRC01gl_Q39LH _comL
VRC01_L39QH _del103L
VRC01gl_L104VL_E105DL
VRC01gl_Q39LH _ins103L
VRC01gl_Q39LH _Y91FH
VRC01gl
I
VRC01gl_comL
VRC01gl_Q39LH
CDR1
CDR2
DE-loop
CDR3
VRC01gl_Q39LH _comL
E
J
VRC01
VRC01_del103L
CDR1
CDR2
DE-loop
CDR3
VRC01_L39QH
VRC01_L39QH _del103L
Figure S2. Modulation of antibody conformation and flexibility by somatic hypermutations at the VH-VL and elbow interface residues.
Dynamics of the HL angle over 1ms MD simulation. Five repeats of 1ms MD simulation were performed for each antibody variant. Non-converged MD runs were excluded.
Dynamics of the elbow angle over 1ms MD simulation. Five repeats of 1ms MD simulation were performed for each antibody variant. Non-converged MD runs were excluded.
-(H) MD simulation sampled VH-VL conformations of the VRC01gl and VRC01 variants. The VH-VL conformation was measured using the five ABangle parameters. All MD repeats were combined for each variant.
(I) Root mean squire flexibility (RMSF) of VRC01gl variants measured from MD simulation.
(J) Root mean squire flexibility of VRC01 variants measured from MD simulation.

## Slide 6
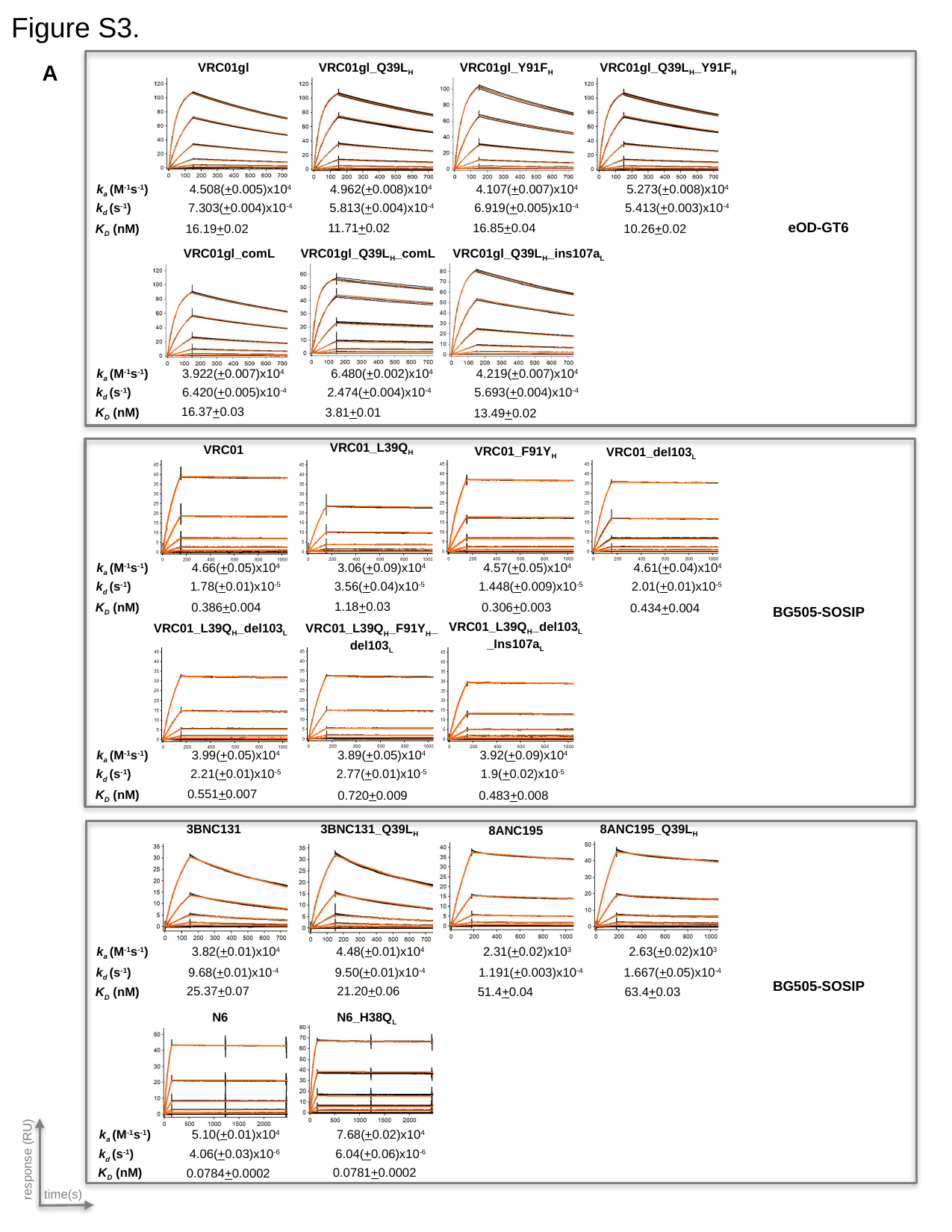

# Figure S3.
A
VRC01gl
VRC01gl_Q39LH
VRC01gl_Y91FH
VRC01gl_Q39LH_Y91FH
ka (M-1s-1)
4.508(+0.005)x104
7.303(+0.004)x10-4
16.19+0.02
4.962(+0.008)x104
5.813(+0.004)x10-4
11.71+0.02
4.107(+0.007)x104
6.919(+0.005)x10-4
16.85+0.04
5.273(+0.008)x104
5.413(+0.003)x10-4
10.26+0.02
kd (s-1)
eOD-GT6
KD (nM)
VRC01gl_comL
VRC01gl_Q39LH_comL
VRC01gl_Q39LH_ins107aL
ka (M-1s-1)
3.922(+0.007)x104
6.420(+0.005)x10-4
16.37+0.03
6.480(+0.002)x104
2.474(+0.004)x10-4
3.81+0.01
4.219(+0.007)x104
5.693(+0.004)x10-4
13.49+0.02
kd (s-1)
KD (nM)
VRC01_L39QH
VRC01
VRC01_F91YH
VRC01_del103L
ka (M-1s-1)
kd (s-1)
KD (nM)
4.66(+0.05)x104
1.78(+0.01)x10-5
0.386+0.004
3.06(+0.09)x104
3.56(+0.04)x10-5
1.18+0.03
4.57(+0.05)x104
1.448(+0.009)x10-5
0.306+0.003
4.61(+0.04)x104
2.01(+0.01)x10-5
0.434+0.004
BG505-SOSIP
VRC01_L39QH_del103L_Ins107aL
VRC01_L39QH_del103L
VRC01_L39QH_F91YH_del103L
ka (M-1s-1)
kd (s-1)
KD (nM)
3.99(+0.05)x104
2.21(+0.01)x10-5
0.551+0.007
3.89(+0.05)x104
2.77(+0.01)x10-5
0.720+0.009
3.92(+0.09)x104
1.9(+0.02)x10-5
0.483+0.008
3BNC131_Q39LH
3BNC131
8ANC195_Q39LH
8ANC195
ka (M-1s-1)
kd (s-1)
KD (nM)
3.82(+0.01)x104
9.68(+0.01)x10-4
25.37+0.07
4.48(+0.01)x104
9.50(+0.01)x10-4
21.20+0.06
2.31(+0.02)x103
1.191(+0.003)x10-4
51.4+0.04
2.63(+0.02)x103
1.667(+0.05)x10-4
63.4+0.03
BG505-SOSIP
N6
N6_H38QL
ka (M-1s-1)
5.10(+0.01)x104
4.06(+0.03)x10-6
0.0784+0.0002
7.68(+0.02)x104
6.04(+0.06)x10-6
0.0781+0.0002
kd (s-1)
response (RU)
KD (nM)
time(s)

## Slide 7
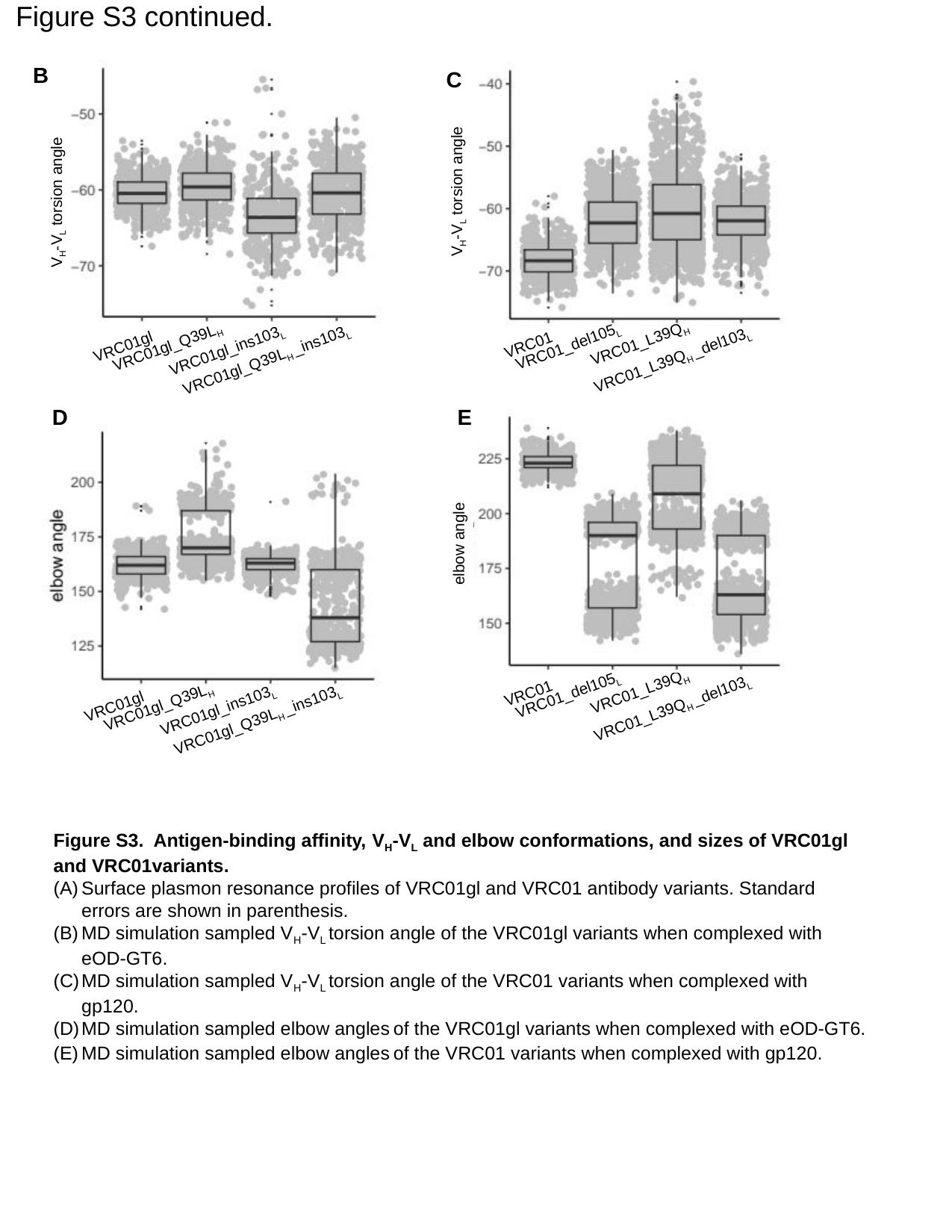

# Figure S3 continued.
B
C
VH-VL torsion angle
VH-VL torsion angle
VRC01_L39QH
VRC01
VRC01_del105L
VRC01gl
VRC01gl_Q39LH
VRC01gl_ins103L
VRC01_L39QH _del103L
VRC01gl_Q39LH _ins103L
E
D
elbow angle
VRC01_L39QH
VRC01
VRC01_del105L
VRC01gl
VRC01gl_Q39LH
VRC01_L39QH _del103L
VRC01gl_ins103L
VRC01gl_Q39LH _ins103L
Figure S3. Antigen-binding affinity, VH-VL and elbow conformations, and sizes of VRC01gl and VRC01variants.
Surface plasmon resonance profiles of VRC01gl and VRC01 antibody variants. Standard errors are shown in parenthesis.
MD simulation sampled VH-VL torsion angle of the VRC01gl variants when complexed with eOD-GT6.
MD simulation sampled VH-VL torsion angle of the VRC01 variants when complexed with gp120.
MD simulation sampled elbow angles of the VRC01gl variants when complexed with eOD-GT6.
MD simulation sampled elbow angles of the VRC01 variants when complexed with gp120.

## Slide 8
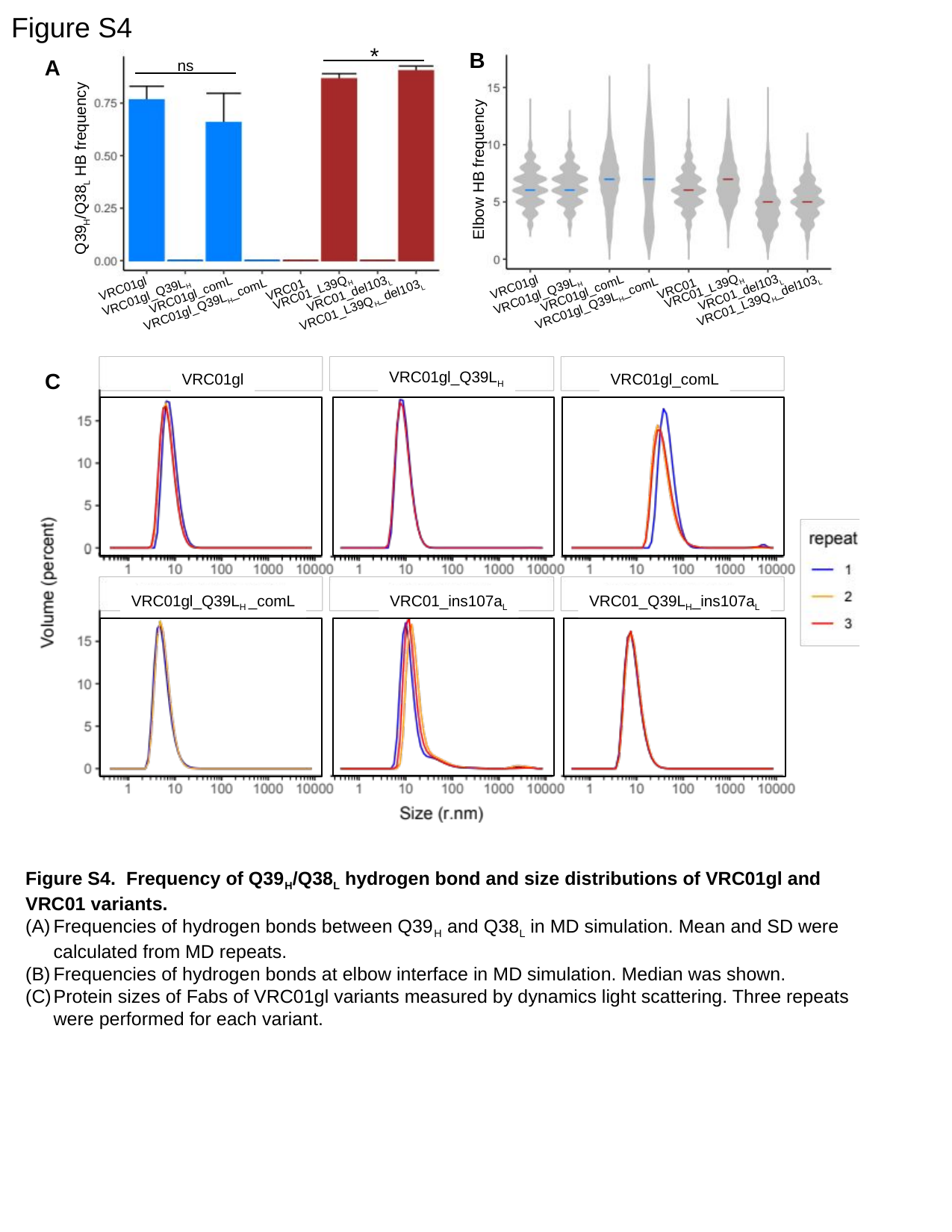

# Figure S4
*
B
A
ns
Q39H/Q38L HB frequency
Elbow HB frequency
VRC01gl
VRC01
VRC01gl
VRC01
VRC01_L39QH
VRC01_L39QH
VRC01_del103L
VRC01_del103L
VRC01gl_comL
VRC01gl_comL
VRC01gl_Q39LH
VRC01gl_Q39LH
VRC01_L39QH_del103L
VRC01gl_Q39LH_comL
VRC01_L39QH_del103L
VRC01gl_Q39LH_comL
VRC01gl_Q39LH
C
VRC01gl_comL
VRC01gl
VRC01gl_Q39LH _comL
VRC01_ins107aL
VRC01_Q39LH_ins107aL
Figure S4. Frequency of Q39H/Q38L hydrogen bond and size distributions of VRC01gl and VRC01 variants.
Frequencies of hydrogen bonds between Q39H and Q38L in MD simulation. Mean and SD were calculated from MD repeats.
Frequencies of hydrogen bonds at elbow interface in MD simulation. Median was shown.
Protein sizes of Fabs of VRC01gl variants measured by dynamics light scattering. Three repeats were performed for each variant.

## Slide 9
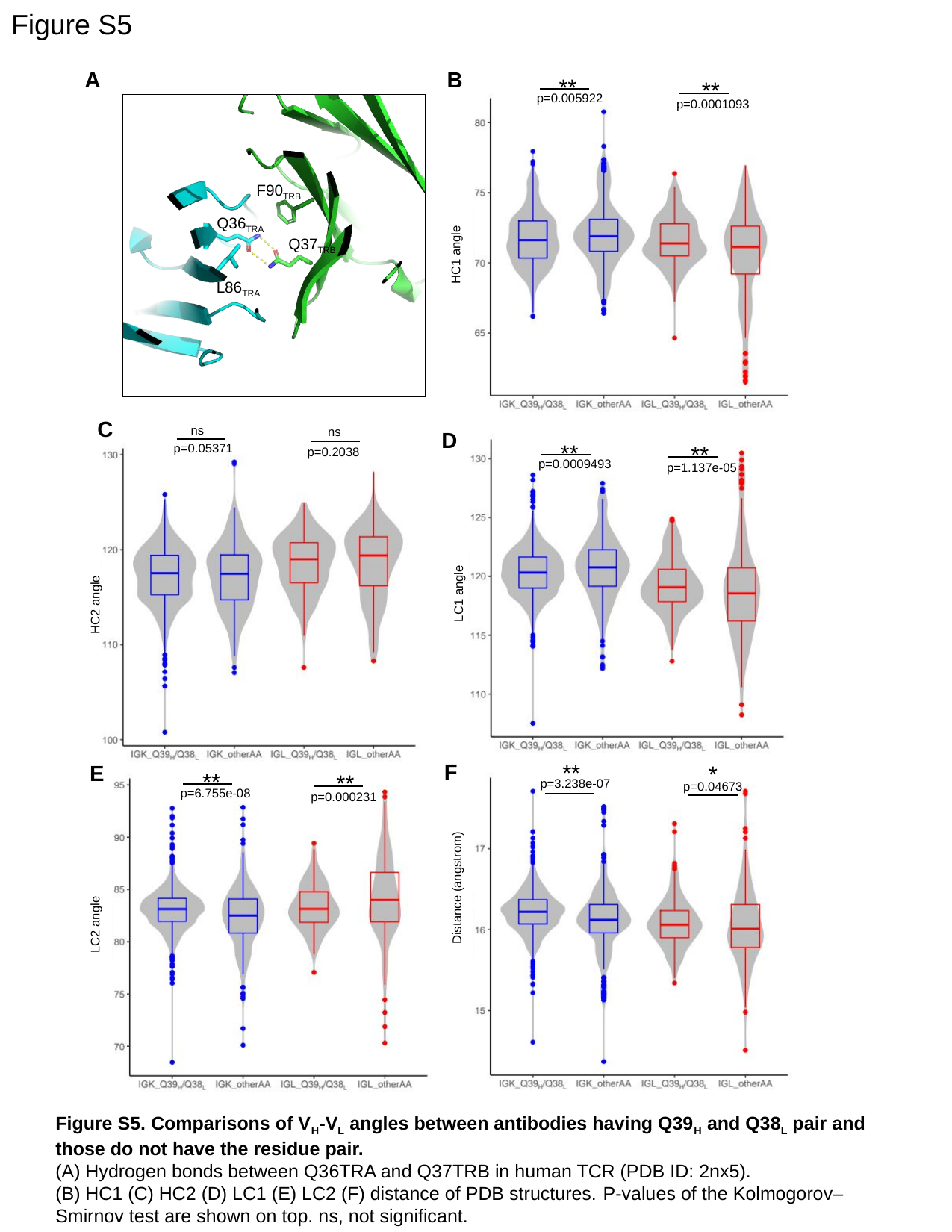

# Figure S5
A
B
**
**
p=0.005922
p=0.0001093
F90TRB
Q36TRA
Q37TRB
HC1 angle
L86TRA
C
ns
ns
D
**
p=0.05371
**
p=0.2038
p=0.0009493
p=1.137e-05
LC1 angle
HC2 angle
F
**
*
E
**
**
p=3.238e-07
p=0.04673
p=6.755e-08
p=0.000231
Distance (angstrom)
LC2 angle
Figure S5. Comparisons of VH-VL angles between antibodies having Q39H and Q38L pair and those do not have the residue pair.
(A) Hydrogen bonds between Q36TRA and Q37TRB in human TCR (PDB ID: 2nx5).
(B) HC1 (C) HC2 (D) LC1 (E) LC2 (F) distance of PDB structures. P-values of the Kolmogorov–Smirnov test are shown on top. ns, not significant.

## Slide 10
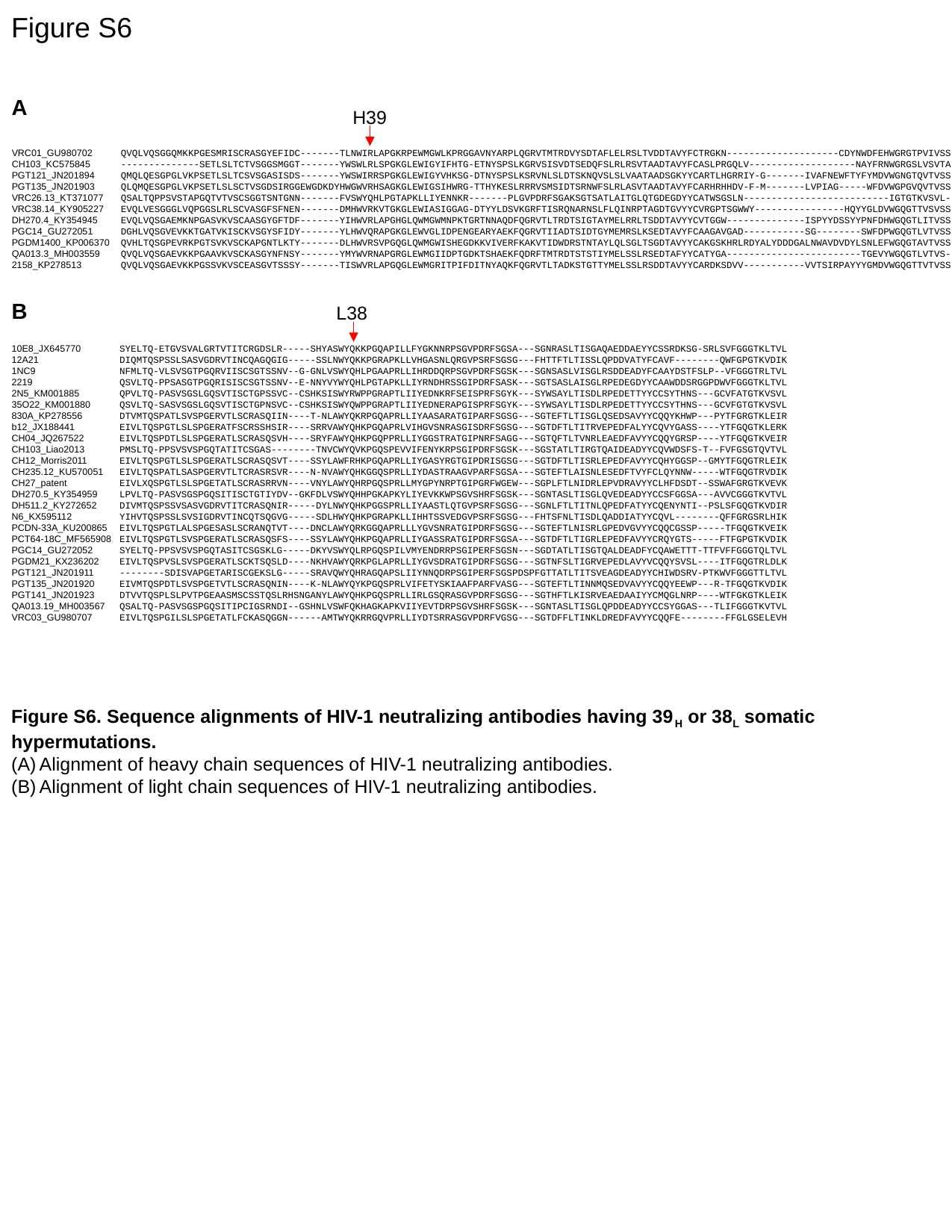

# Figure S6
A
H39
QVQLVQSGGQMKKPGESMRISCRASGYEFIDC-------TLNWIRLAPGKRPEWMGWLKPRGGAVNYARPLQGRVTMTRDVYSDTAFLELRSLTVDDTAVYFCTRGKN--------------------CDYNWDFEHWGRGTPVIVSS
--------------SETLSLTCTVSGGSMGGT-------YWSWLRLSPGKGLEWIGYIFHTG-ETNYSPSLKGRVSISVDTSEDQFSLRLRSVTAADTAVYFCASLPRGQLV-------------------NAYFRNWGRGSLVSVTA
QMQLQESGPGLVKPSETLSLTCSVSGASISDS-------YWSWIRRSPGKGLEWIGYVHKSG-DTNYSPSLKSRVNLSLDTSKNQVSLSLVAATAADSGKYYCARTLHGRRIY-G-------IVAFNEWFTYFYMDVWGNGTQVTVSS
QLQMQESGPGLVKPSETLSLSCTVSGDSIRGGEWGDKDYHWGWVRHSAGKGLEWIGSIHWRG-TTHYKESLRRRVSMSIDTSRNWFSLRLASVTAADTAVYFCARHRHHDV-F-M-------LVPIAG-----WFDVWGPGVQVTVSS
QSALTQPPSVSTAPGQTVTVSCSGGTSNTGNN-------FVSWYQHLPGTAPKLLIYENNKR-------PLGVPDRFSGAKSGTSATLAITGLQTGDEGDYYCATWSGSLN--------------------------IGTGTKVSVL-
EVQLVESGGGLVQPGGSLRLSCVASGFSFNEN-------DMHWVRKVTGKGLEWIASIGGAG-DTYYLDSVKGRFTISRQNARNSLFLQINRPTAGDTGVYYCVRGPTSGWWY----------------HQYYGLDVWGQGTTVSVSS
EVQLVQSGAEMKNPGASVKVSCAASGYGFTDF-------YIHWVRLAPGHGLQWMGWMNPKTGRTNNAQDFQGRVTLTRDTSIGTAYMELRRLTSDDTAVYYCVTGGW--------------ISPYYDSSYYPNFDHWGQGTLITVSS
DGHLVQSGVEVKKTGATVKISCKVSGYSFIDY-------YLHWVQRAPGKGLEWVGLIDPENGEARYAEKFQGRVTIIADTSIDTGYMEMRSLKSEDTAVYFCAAGAVGAD-----------SG--------SWFDPWGQGTLVTVSS
QVHLTQSGPEVRKPGTSVKVSCKAPGNTLKTY-------DLHWVRSVPGQGLQWMGWISHEGDKKVIVERFKAKVTIDWDRSTNTAYLQLSGLTSGDTAVYYCAKGSKHRLRDYALYDDDGALNWAVDVDYLSNLEFWGQGTAVTVSS
QVQLVQSGAEVKKPGAAVKVSCKASGYNFNSY-------YMYWVRNAPGRGLEWMGIIDPTGDKTSHAEKFQDRFTMTRDTSTSTIYMELSSLRSEDTAFYYCATYGA------------------------TGEVYWGQGTLVTVS-
QVQLVQSGAEVKKPGSSVKVSCEASGVTSSSY-------TISWVRLAPGQGLEWMGRITPIFDITNYAQKFQGRVTLTADKSTGTTYMELSSLRSDDTAVYYCARDKSDVV-----------VVTSIRPAYYYGMDVWGQGTTVTVSS
VRC01_GU980702
CH103_KC575845
PGT121_JN201894
PGT135_JN201903
VRC26.13_KT371077
VRC38.14_KY905227
DH270.4_KY354945
PGC14_GU272051
PGDM1400_KP006370
QA013.3_MH003559
2158_KP278513
B
L38
SYELTQ-ETGVSVALGRTVTITCRGDSLR-----SHYASWYQKKPGQAPILLFYGKNNRPSGVPDRFSGSA---SGNRASLTISGAQAEDDAEYYCSSRDKSG-SRLSVFGGGTKLTVL
DIQMTQSPSSLSASVGDRVTINCQAGQGIG-----SSLNWYQKKPGRAPKLLVHGASNLQRGVPSRFSGSG---FHTTFTLTISSLQPDDVATYFCAVF--------QWFGPGTKVDIK
NFMLTQ-VLSVSGTPGQRVIISCSGTSSNV--G-GNLVSWYQHLPGAAPRLLIHRDDQRPSGVPDRFSGSK---SGNSASLVISGLRSDDEADYFCAAYDSTFSLP--VFGGGTRLTVL
QSVLTQ-PPSASGTPGQRISISCSGTSSNV--E-NNYVYWYQHLPGTAPKLLIYRNDHRSSGIPDRFSASK---SGTSASLAISGLRPEDEGDYYCAAWDDSRGGPDWVFGGGTKLTVL
QPVLTQ-PASVSGSLGQSVTISCTGPSSVC--CSHKSISWYRWPPGRAPTLIIYEDNKRFSEISPRFSGYK---SYWSAYLTISDLRPEDETTYYCCSYTHNS---GCVFATGTKVSVL
QSVLTQ-SASVSGSLGQSVTISCTGPNSVC--CSHKSISWYQWPPGRAPTLIIYEDNERAPGISPRFSGYK---SYWSAYLTISDLRPEDETTYYCCSYTHNS---GCVFGTGTKVSVL
DTVMTQSPATLSVSPGERVTLSCRASQIIN----T-NLAWYQKRPGQAPRLLIYAASARATGIPARFSGSG---SGTEFTLTISGLQSEDSAVYYCQQYKHWP---PYTFGRGTKLEIR
EIVLTQSPGTLSLSPGERATFSCRSSHSIR----SRRVAWYQHKPGQAPRLVIHGVSNRASGISDRFSGSG---SGTDFTLTITRVEPEDFALYYCQVYGASS----YTFGQGTKLERK
EIVLTQSPDTLSLSPGERATLSCRASQSVH----SRYFAWYQHKPGQPPRLLIYGGSTRATGIPNRFSAGG---SGTQFTLTVNRLEAEDFAVYYCQQYGRSP----YTFGQGTKVEIR
PMSLTQ-PPSVSVSPGQTATITCSGAS--------TNVCWYQVKPGQSPEVVIFENYKRPSGIPDRFSGSK---SGSTATLTIRGTQAIDEADYYCQVWDSFS-T--FVFGSGTQVTVL
EIVLTQSPGTLSLSPGERATLSCRASQSVT----SSYLAWFRHKPGQAPRLLIYGASYRGTGIPDRISGSG---SGTDFTLTISRLEPEDFAVYYCQHYGGSP--GMYTFGQGTRLEIK
EIVLTQSPATLSASPGERVTLTCRASRSVR----N-NVAWYQHKGGQSPRLLIYDASTRAAGVPARFSGSA---SGTEFTLAISNLESEDFTVYFCLQYNNW-----WTFGQGTRVDIK
EIVLXQSPGTLSLSPGETATLSCRASRRVN----VNYLAWYQHRPGQSPRLLMYGPYNRPTGIPGRFWGEW---SGPLFTLNIDRLEPVDRAVYYCLHFDSDT--SSWAFGRGTKVEVK
LPVLTQ-PASVSGSPGQSITISCTGTIYDV--GKFDLVSWYQHHPGKAPKYLIYEVKKWPSGVSHRFSGSK---SGNTASLTISGLQVEDEADYYCCSFGGSA---AVVCGGGTKVTVL
DIVMTQSPSSVSASVGDRVTITCRASQNIR-----DYLNWYQHKPGGSPRLLIYAASTLQTGVPSRFSGSG---SGNLFTLTITNLQPEDFATYYCQENYNTI--PSLSFGQGTKVDIR
YIHVTQSPSSLSVSIGDRVTINCQTSQGVG-----SDLHWYQHKPGRAPKLLIHHTSSVEDGVPSRFSGSG---FHTSFNLTISDLQADDIATYYCQVL--------QFFGRGSRLHIK
EIVLTQSPGTLALSPGESASLSCRANQTVT----DNCLAWYQRKGGQAPRLLLYGVSNRATGIPDRFSGSG---SGTEFTLNISRLGPEDVGVYYCQQCGSSP-----TFGQGTKVEIK
EIVLTQSPGTLSVSPGERATLSCRASQSFS----SSYLAWYQHKPGQAPRLLIYGASSRATGIPDRFSGSA---SGTDFTLTIGRLEPEDFAVYYCRQYGTS-----FTFGPGTKVDIK
SYELTQ-PPSVSVSPGQTASITCSGSKLG-----DKYVSWYQLRPGQSPILVMYENDRRPSGIPERFSGSN---SGDTATLTISGTQALDEADFYCQAWETTT-TTFVFFGGGTQLTVL
EIVLTQSPVSLSVSPGERATLSCKTSQSLD----NKHVAWYQRKPGLAPRLLIYGVSDRATGIPDRFSGSG---SGTNFSLTIGRVEPEDLAVYVCQQYSVSL----ITFGQGTRLDLK
--------SDISVAPGETARISCGEKSLG-----SRAVQWYQHRAGQAPSLIIYNNQDRPSGIPERFSGSPDSPFGTTATLTITSVEAGDEADYYCHIWDSRV-PTKWVFGGGTTLTVL
EIVMTQSPDTLSVSPGETVTLSCRASQNIN----K-NLAWYQYKPGQSPRLVIFETYSKIAAFPARFVASG---SGTEFTLTINNMQSEDVAVYYCQQYEEWP---R-TFGQGTKVDIK
DTVVTQSPLSLPVTPGEAASMSCSSTQSLRHSNGANYLAWYQHKPGQSPRLLIRLGSQRASGVPDRFSGSG---SGTHFTLKISRVEAEDAAIYYCMQGLNRP----WTFGKGTKLEIK
QSALTQ-PASVSGSPGQSITIPCIGSRNDI--GSHNLVSWFQKHAGKAPKVIIYEVTDRPSGVSHRFSGSK---SGNTASLTISGLQPDDEADYYCCSYGGAS---TLIFGGGTKVTVL
EIVLTQSPGILSLSPGETATLFCKASQGGN------AMTWYQKRRGQVPRLLIYDTSRRASGVPDRFVGSG---SGTDFFLTINKLDREDFAVYYCQQFE--------FFGLGSELEVH
10E8_JX645770
12A21
1NC9
2219
2N5_KM001885
35O22_KM001880
830A_KP278556
b12_JX188441
CH04_JQ267522
CH103_Liao2013
CH12_Morris2011
CH235.12_KU570051
CH27_patent
DH270.5_KY354959
DH511.2_KY272652
N6_KX595112
PCDN-33A_KU200865
PCT64-18C_MF565908
PGC14_GU272052
PGDM21_KX236202
PGT121_JN201911
PGT135_JN201920
PGT141_JN201923
QA013.19_MH003567
VRC03_GU980707
Figure S6. Sequence alignments of HIV-1 neutralizing antibodies having 39H or 38L somatic hypermutations.
Alignment of heavy chain sequences of HIV-1 neutralizing antibodies.
Alignment of light chain sequences of HIV-1 neutralizing antibodies.
